# Morphological consequences of artificial cranial deformation: Modularity and integration

**DOI:** 10.1371/journal.pone.0227362

**Published:** 2020-01-24

**Authors:** Thomas A. Püschel, Martin Friess, Germán Manríquez

**Affiliations:** 1 Primate Models for Behavioural Evolution, Institute of Cognitive and Evolutionary Anthropology, University of Oxford, Oxford, United Kingdom; 2 Département Homme et Environnement, Muséum National d'Histoire Naturelle, Paris, France; 3 Instituto de Investigación en Ciencias Odontológicas, Centro de Análisis Cuantitativo en Antropología Dental, Facultad de Odontología, Universidad de Chile, Santiago, Chile; 4 Departamento de Antropología, Facultad de Ciencias Sociales, Universidad de Chile, Santiago, Chile; The Cyprus Institute, CYPRUS

## Abstract

The cranium is an anatomically complex structure. One source of its complexity is due to its modular organization. Cranial modules are distinct and partially independent units that interact substantially during ontogeny thus generating morphological integration. Artificial Cranial Deformation (ACD) occurs when the human skull is intentionally deformed, through the use of different deforming devices applied to the head while it is developing. Hence, ACD provides an interesting example to assess the degree to which biomechanical perturbations of the developing neurocranium impact on the degree of morphological integration in the skull as a whole. The main objective of this study was to assess how ACD affects the morphological integration of the skull. This was accomplished by comparing a sample of non-deformed crania and two sets of deformed crania (i.e. antero-posterior and oblique). Both developmental and static modularity and integration were assessed through Generalized Procrustes Analysis by considering the symmetric and asymmetric components of variation in adults, using 3D landmark coordinates as raw data. The presence of two developmental modules (i.e. viscero and neurocranium) in the skull was tested. Then, in order to understand how ACD affects morphological integration, the covariation pattern between the neuro and viscerocranium was examined in antero-posterior, oblique and non-deformed cranial categories using Partial Least-Squares. The main objective of this study was to assess how ACD affects the morphological integration of the skull. This was accomplished by comparing a sample of deformed (i.e. antero-posterior and oblique) and non-deformed crania. Hence, differences in integration patterns were compared between groups. The obtained results support the modular organization of the human skull in the two analyzed modules. The integration analyses show that the oblique ACD style differentially affects the static morphological integration of the skull by increasing the covariance between neuro and viscerocranium in a more constrained way than in antero-posterior and non-deformed skulls. In addition, the antero-posterior ACD style seems to affect the developmental integration of the skull by directing the covariation pattern in a more defined manner as compared to the other cranial categories.

## Introduction

There is longstanding scientific tradition that has analyzed the complex nature of human skull using developmental [1], functional [2] and evolutionary [3] approaches, among others. The diverse components of the skulls are integrated with respect to each other because they have developed, functioned and evolved jointly [4]. The morphological integration of the skull is an inexorable process because cranial components are packed together, they have a common developmental background, functional demands exerting reiterative pressures and all the cranial portions share an evolutionary history [5,6]. This integration among different components of the cranium can occur through many biological processes such as hormonal influences, pleiotropy, scaling, regional interactions, among others [7]. However it is important to notice that even though integration is a common biological phenomenon manifested at different levels [7–9], the underlying causes generating it are in most situations not directly observable. Although a recently proposed model-bound approach for understanding morphological evolution of the human skull considers not only statistical analysis of form, but also quantitative genetics and explicit evolutionary hypotheses, like neutral theory [10], morphological integration is typically studied by analyzing the covariation among morphological traits [11]. Nonetheless, this integration is not absolute but organized in units that are relatively independent while participating to generate a structure that act as functional whole. This fact has been recognized for decades [12–16]. These independent units are nevertheless integrated internally, and are operationally known as modules [7]. Even though the majority of the studies on modularity and integration have focused on variation among individuals within populations, there are more levels of variation that exhibit modularity and integration [9], deriving from distinct sources such as genetic variation, phenotypic plasticity, fluctuating asymmetry, evolutionary change, among others [10,17].

Morphological integration and modularity has been studied in skulls by defining modules based in its differential embryological origin: the viscerocranium -derived from branchial arches-, and the neurocranium—derived from neural crest and paraxial mesoderm cells [4,18–26]. There is some evidence that the human skull has a modular behavior divided in two main modules based in its differential embryological origin: the viscerocranium or facial skeleton and the neurocranium or braincase [24,25,27–31]. Based on a similar criterion [32], the latter has been also divided in two additional regions: i.e. the basicranium with endochondral ossification from a cartilaginous mesodermic precursor (chondrocranium) [3,33–37], and the calvaria whose bones derive from the desmocranium, which has an intramembranous ossification from paraxial mesoderm and neural crests cells [38]. In fact, different studies have shown that the patterns of covariation and correlation between different parts of the cranium are partially independent, thus suggesting that they behave as partially independent units [15,39–42]. On the other hand, it has been also suggested that the development of the skull is extremely integrated both functionally and ontogenetically, so any mechanic perturbation could affect not only the immediately adjacent tissues but also other structures that covary with them [43]. For example, children with cartilage growth defects in the cranial base often develop abnormal calvarial morphologies, malocclusion and concave faces [43,44]. Likewise, children with craniosynostoses usually develop malformations of the skeletal face and base [45,46].

In the present study artificial cranial deformation (ACD) is used as a proxy for assessing the morphological integration and modularity of the human skull. ACD consists in the modification of the magnitude and direction of the normal vectors of growth and development of the skull, by using compressive forces generated by deforming devices during the early years of post-natal life [47]. It is a cultural practice which modifies the newborn’s natural head shape to achieve a certain desired morphology, being since long considered either as an identity symbol and/or social status marker in different populations around the world [48–50]. Depending on the particular tradition, ACD comprises the application of different methods, such as bandages, boards, stones, pads, or a combination of these to the neonate skull [48]. Ethnographic evidence shows even that intentional head deformation can be even achieved by manual modification [51], and has been carried out not only in pre-historical but in historical [52], and present time societies too [53]. This cranial modification practice was widely distributed both geographically and temporally [1,49,51,54–56]. Despite findings about the presence of ACD in Central Asia [57–59], in Near and Middle East [60–62], in Eastern [63–67], and Western Europe [68–71], ACD exhibits particularly high frequencies in the Americas as exemplified in [72–74], especially in the South-Central Andes [1,51,55,73,75–80]. Regarding the effect that ACD has on the skull the available evidence shows that ACD is not confined to the calvaria but also affects the cranial base. Despite the fact that deforming devices are mostly placed on the neurocranium [47,81–84], as a result of ACD practice cranial base decreases its normal angle and increases the anterior-inferior projection of the viscerocranium. It seems that ACD influences the complete skull by affecting its globularity, which could mean that the cranial vault behaves as a highly constrained trait [85]. Under certain circumstances ACD could even cause the death of the newborn, as is evidenced by the pathognomonic periosteal reaction at the occipital and parietals bones found in the artificially deformed skull of a one year old children from a late Inca burial [86].

Considering that the ACD constrains the skull’s normal ontogenetic growth direction, it would be expected that the expansion of the encephalon will continue generating internal pressures that will be redirected towards those areas of the cranial vault that are not restricted by the application of the deforming device. In fact, it has been shown that ACD typically generates some amount of compensatory growth in the sutures [87,88]. It seems likely that when the deforming device is applied for a long period, the internal pressures within the skull will continue, however they will be more focused to other cranial regions. This can probably increase or decrease the covariation between the different cranial regions, modifying the pattern of shape/size variation of other bones like the zygomatic bone [89], the shape and volume of craniofacial cavities [90,91], and the basicranial, and the relative position of the mandibular fossae [92]. Therefore, ACD provides an interesting example to assess the degree to which biomechanical perturbations of the developing neurocranium impact on the degree of morphological integration in the cranium as a whole. The null hypotheses that lead this research are:

**h0a:** The human skull does not exhibit a modular structure corresponding to the viscero and neurocranium.
**h0b:** ACD, independently of the type of deformation, does not change the degree of morphological integration of the skull compared with non-deformed individuals.

## Materials and method

**Fig 1:**
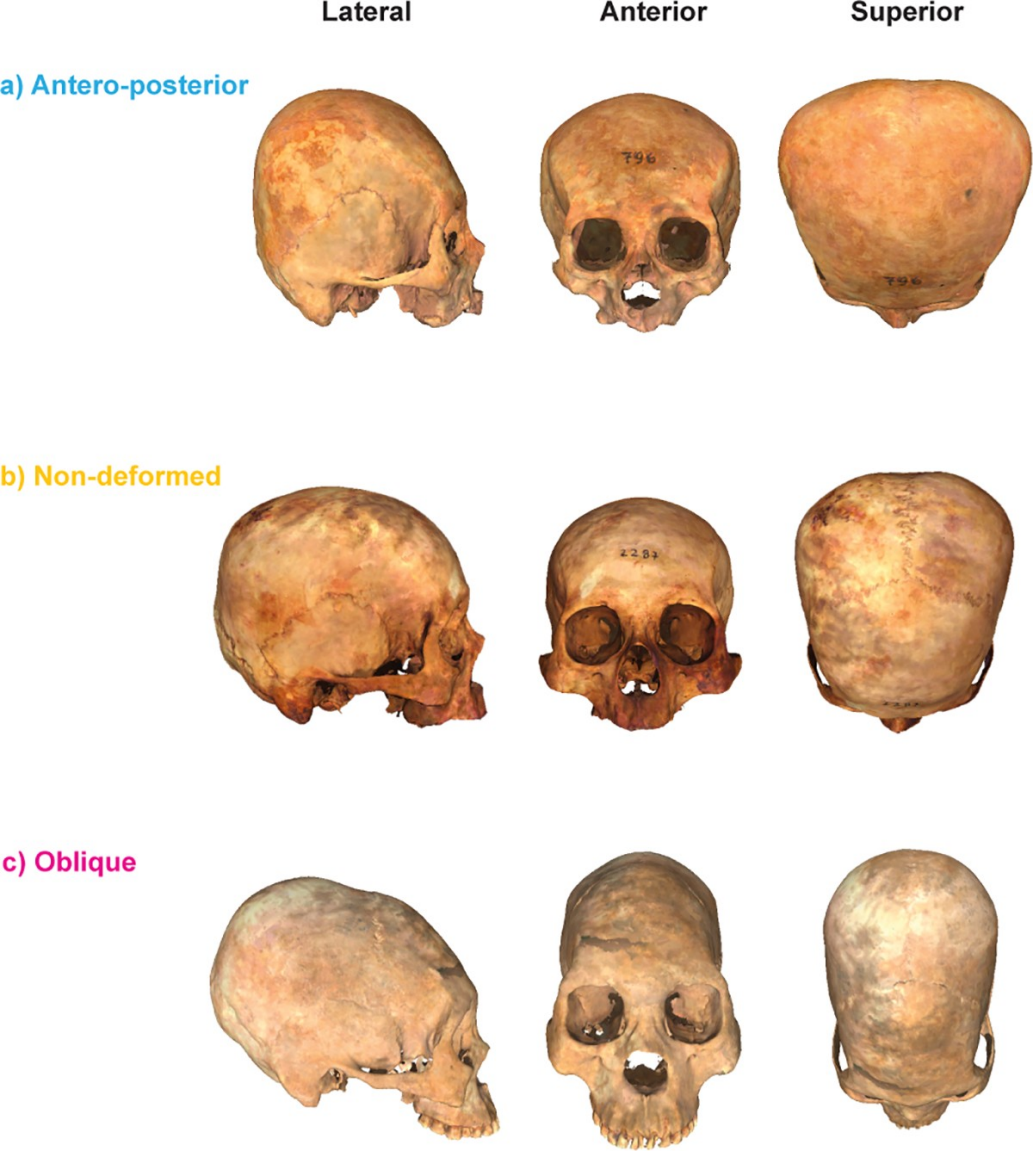
Representative 3D models. 3D surfaces of the different cranial categories analyzed in the present paper and their corresponding anatomical views.

The sample comprised 269 archaeological skulls from Northern Chile (Table 1). Individual numbers and complete repository information, including museum name and geographic location are provided in S1 Table. No permits were required for the described study, which complied with all relevant regulations. The skulls were scanned using a NextEngine Desktop 3D Scanner (model 2020i; Santa Monica, CA) in two different positions, each of them composed by ten divisions to allow a complete three-dimensional (3D) reconstruction. The number of divisions controls the degree of rotation between scans and the total number of scans, so each division correspond to 36° of the total rotation. 3D models were generated by trimming the unwanted artifacts, aligning the two different positions and fusing the two surfaces of each skull using both Meshlab v.1.3.3 (http://meshlab.sourceforge.net/) and ScanStudio^™^ (http://www.nextengine.com/). All the individuals analyzed in this study were adults. The following inclusion criteria were followed: third molar eruption and the fusion of the spheno-occipital synchondrosis. Deformed skulls were classified as antero-posterior or oblique deformations based on a simplified version of the classification proposed by [93] (Fig 1). Antero-posteriorly deformed skulls were characterized by an antero-posterior vault compression, resulting in the flattening of the frontal and occipital bones, alongside a lateral expansion of the head. On the other hand, oblique skulls were defined according to their conical and lengthened cranial vault, with a relatively narrowed medial-lateral dimension.

**Table 1:** Sample used in the present study.

Origin	Antero-posterior	Non-deformed	Oblique
**Caspana**	5	5	16
**Catarpe 2**	4	31	10
**Chorrillos**	0	0	20
**Chunchuri**	8	4	5
**Coyo Oriente**	8	10	2
**Coyo 3**	3	5	0
**Larache**	4	11	0
**Quitor 5**	0	2	1
**Quitor 6**	4	3	2
**Sequitor Alambrado**	0	5	1
**Sequitor Alambrado Oriente**	4	5	0
**Solcor**	8	3	1
**Solcor 3**	4	8	5
**Solor 3**	1	2	3
**Tchecar**	12	9	1
**Tchilimoya**	4	11	4
**Toconao**	0	3	0
**Toconao Oriente**	2	1	1
**Toconce**	1	0	2
**Topater**	2	0	3
	**74**	**118**	**77**

Origin of the analyzed sample (archaeological site), and type of artificial cranial deformation. Skulls are housed at Museo Arqueológico R.P. Gustavo Le Paige, San Pedro de Atacama

Chile, excluding Chorrillos (Corporación Cultural y de Turismo, Calama, Chile), and

Chunchuri (Musée de l’Homme, Paris, France).

**Fig 2:**
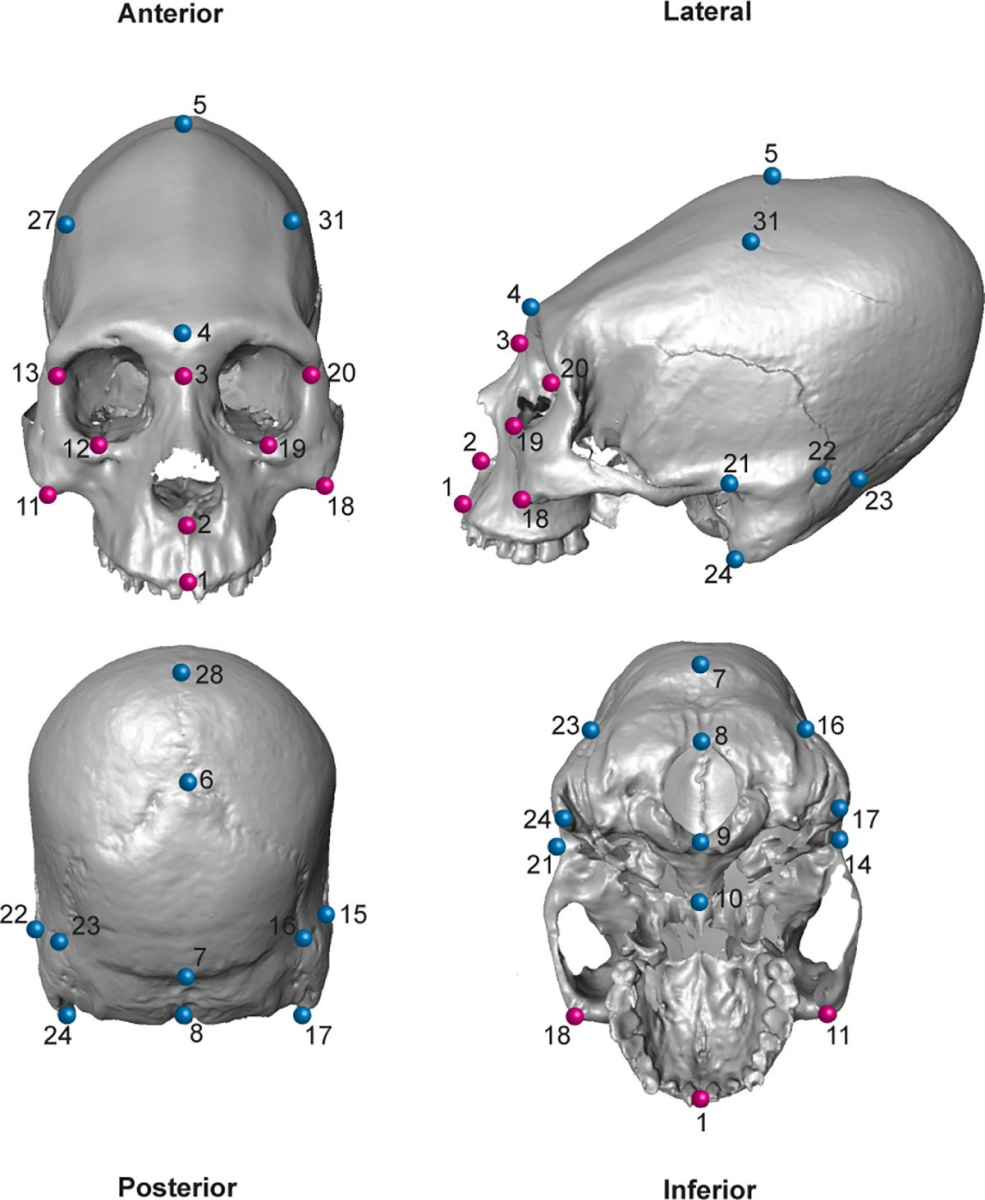
Landmarks used to carry out the GM analyses. An oblique artificially deformed skull from the archaeological site of Chorrillos is shown (blue = neurocranium; fuchsia = viscerocranium).

This first step was followed by a geometric morphometrics generalized Procrustes superimposition of landmark data. This branch of shape analysis has been usually understood as the quantitative study of shape and its covariates [94]. Landmark acquisition was carried out by one of us (TP) in Landmark Editor v.3.6 software (IDAV) [95] by collecting 31 homologous and well-defined 3D points using the nomenclature of [96] (Table 2; Fig 2). These raw coordinates are provided in S1 Table. Geometric morphometrics and statistical analysis were carried out in MorphoJ v. 1.06d [97] and the R package ‘geomorph’ [98]. One surface file was warped using Landmark Editor v.3.6 [95] in order to visualize shape changes depending on the applied analysis. Measurement error (ME) has a critical importance when performing morphometric analyses, therefore to assess it, a sub-sample of 170 skulls were digitized twice and compared via a Procrustes ANOVA [99].

**Table 2:** Landmarks used in the present study.

Number	Name	Definition	Module
**1**	Prostion (pr)	The most anterior point on the alveolar ridge of the maxilla between the central incisors in the median sagittal plane.	Viscerocranium
**2**	Nasospinale (ns)	The lowest point of the lower border of the piriform aperture, projected into the median sagittal plane.	Viscerocranium
**3**	Nasion (n)	Crossing point of the frontonasal suture with the median sagittal.	Viscerocranium
**4**	Glabella (g)	Most anterior point, in the mediansagittal, betwen the superciliary arches	Neurocranium
**5**	Bregma (b)	The point at which the sagittal suture meets the coronal suture.	Neurocranium
**6**	Lambda (l)	The point at which the two parts of the lambdoidal suture meet the sagittal suture.	Neurocranium
**7**	Inion (i)	The point in the median sagittal plane, in which the two superior nuchal lines join.	Neurocranium
**8**	Opistion (o)	The point at which the posterior border of the foramen magnum is cut by the medial sagittal plane.	Neurocranium
**9**	Basion (ba)	The point at which the anterior margin of the foramen magnum is cross by the median sagittal plane.	Neurocranium
**10**	Sphenobasion (sphba)	Intersection of the sphenooccipitalsynchondrosis with the median sagittal plane.	Neurocranium
**11,18**	Zygomaxillare (rzm)	Lowest point of the right zygomaticomaxillary suture	Viscerocranium
**12, 19**	Zygoorbitale (rzo)	Upper point of the right zygomaticomaxillary suture.	Viscerocranium
**13, 20**	Frontomalare orbitale (rfmo)	The only point on the lateral right orbital rim, at which it is cross by the frontozygomatic suture.	Viscerocranium
**14, 21**	Auriculare (rau)	The point that is perpendicular to the center of the right porus acusticus externus located on the zygomatic root.	Neurocranium
**15, 22**	Entomion (ren)	The point at which the right squamous suture passes into the right parietomastoidea suture.	Neurocranium
**16, 23**	Asterion (rast)	The point at which the right lambdoidea, occipitomastoid and parieto-mastoid the sutures meet.	Neurocranium
**17, 24**	Mastoideale (rms)	The most inferior point of the right mastoid process.	Neurocranium
**25, 29**	Krotaphion (rk)	The posterior point of the right sphenoparietalis suture	Neurocranium
**26, 30**	Sphenion (rsphn)	The anterior point of the rigth sphenoparietalis suture	Neurocranium
**27, 31**	Stephanion (rst)	The point where the coronal suture meets the right temporal line	Neurocranium
**28**	Obelion (ob)	The point between a transverse line connecting the two parietal foramina and the sagittal suture.	Neurocranium

Number, name, definition, and location of the landmarks used for carrying out the geometric morphometrics workflow.

Human skulls exhibit object symmetry (i.e. the axis of symmetry passes through mid-plane, and hence the left and right halves are mirror images of each other), therefore the recommendations of [100,101] were followed. A generalized Procrustes analysis taking into account object symmetry was performed to remove the differences due rotation, scale and translation. Due to the presence of object symmetry, two separate matrices were generated, representing the symmetric and asymmetric components of variation respectively [101]. The symmetric component represents shape variation among individuals in what could be regarded as a left-right average, thus being useful to study static integration and modularity. On the other hand, the asymmetric component represents the differences between the original and mirrored configurations, being used in the present study to analyze developmental integration and modularity. These two components represent the shape variables that were used in the subsequent analysis. Due to the fact that there is a morphological continuum between deformed and non-deformed skulls (with slightly deformed skull in between), in order to test if it was possible to distinguish between deformed (i.e. antero-posterior and oblique) and non-deformed skulls a canonical variate analysis (CVA) of the symmetric component was performed using the Procrustes coordinates as raw data. The posterior probabilities from the cross-validated CVA are available in S3 Table (Linear Discriminant Analysis using the Procrustes coordinates of the first 11 PCs after a broken-stick model for 3 classes /'Antero-Posterior', 'Non.deformed', 'Oblique'/, Accuracy = 0.8215613, Kappa = 0.7222987). A principal component analysis (PCA) was also carried out in order to quantify skull shape variation. In addition, pairwise PERMANOVA tests with a Holm-Bonferroni correction for multiple comparisons were carried out to test for shape differences between the three cranial categories [102] (i.e., non-deformed, oblique and antero-posterior). The PERMANOVA corresponds to a non-parametric test of significant difference between two or more classes based on a distance measure [103]. In the present work Euclidean distances calculated from the PC scores obtained from the PCA were used as similarity index and 10.000 permutations were carried out. These tests were carried out by adapting the adonis() function from the R package ‘vegan’ v. 2.5–6 to perform multiple comparisons.

As previously mentioned, depending on the processes responsible for integration and modularity, several levels of integration can be distinguished (e.g. static, developmental, evolutionary, among others) [9]. In the present study two main integration levels were analyzed: a) static integration, which is merely the level of variation among individuals in a homogeneous sample, where all specimens are from the same species and ontogenetic stage (this level was analyzed using the symmetric component), and b) developmental, which refers to the fact that morphological traits tend to be associated statistically when they share a specific function and/or a synchronic appearance during embryonic development (this level was analyzed using the asymmetric component) [9].

From a morphometric point of view, modularity is manifested as a strong correlation within the components of a module, versus a weak covariation between modules [7]. There are several methods to statistically test modularity hypotheses (see for example [4] for a review), including the vectorial correlation (RV) coefficient [104], which is a multivariate analogue of a correlation, where the landmark configurations are divided into the hypothetical modules. However, this coefficient has been criticized because it can be adversely affected by the attributes of the data under analysis (i.e. sample size and the number of variables) [105]. Therefore, the covariance ratio (CR) test of modularity was used instead. This test corresponds to a ratio of between to within modules covariances and can range between zero and more than one and has been proposed to overcome some of the problems of RV coefficient [105].

In the present study, two hypothetical modules were proposed based on developmental grounds for the whole sample: the a) viscerocranium and the b) neurocranium. The two proposed modules are parts of the cranium, being consequently spatially adjacent and within the same structure. Therefore, the analysis was restricted to the generation of only spatially contiguous configurations [106]. Based on the same argument, the modularity test was performed using a single Procrustes fit, which automatically considers the information about the connection and spatial arrangement of the subsets [106]. This analysis was carried out both for the symmetric and asymmetric components of variation separately to analyze both static and developmental modularity.

The morphological integration of the skull components was analyzed by means of a partial least-squares analysis (PLS) [107]. According to the available information (cf. Introduction section, this work), deforming devices were normally applied on the occipital, frontal, parietals and temporal bones (although this varied depending on the specific deforming tradition). Consequently, the PLS analysis was carried out to study the covariation patterns between neurocranium and viscerocranium shape variables. The PLS method finds the principal components of covariation between the sets of variables by means of decomposing the covariance matrix of the two blocks into two sets of eigenvectors (one for each set of variables) and eigenvalues, using a singular value decomposition in order to generate a matrix of singular values (the square-roots of the eigenvalues) [107], a matrix of eigenvectors for the first set of variables and the transpose of the matrix of eigenvectors for the second set of variables [108]. The PLS was carried out within the complete landmark configuration, which means that the analysis considered all the existing covariation between the two blocks, including the part related to relative positions, orientations and sizes of the blocks [7]. This approach is well-suited for morphological integration studies that consider the covariation between parts in the context of an entire structure, where the relative arrangement of the components could produce a significant contribution to the covariation patterns, such as in the case of the human cranium [7]. This procedure was repeated separately for each one of the analyzed categories (i.e. non-deformed, antero-posterior and oblique deformed crania) and for both the symmetric and asymmetric components of variation in order to analyze both static and developmental integration. To compare the differences in the covariation patterns between deformed (i.e. antero-posterior and oblique) and non-deformed skulls, pairwise permutation tests of the angular differences between the PLS vectors were estimated [109]. In addition, an angular comparison between the PC’s and the PLS axes was performed to test if the observed patterns of morphological variation (i.e. PC’s) followed a similar trajectory as the observed covariation pattern between the neuro and the viscerocranium (i.e. PLS axes) [110]. In a similar fashion, from all the possible comparisons only a few of them were significant (Symmetric component: θ PC1 v/s PLS1 = 13.361°, P-value <0.00001; Asymmetric component: θ PC1 v/s PLS1 = 34.190°, P-value <0.00001; θ PC2 v/s PLS2 = 49.545°, P-value <0.00001).

Finally, in order to quantify the overall similarity of covariance matrices, matrix correlations and the associated permutation tests were computed, using procedures adapted for geometric morphometrics and object symmetry [7,99,101]. These procedures were carried out to analyze whether the observed patterns of variation were similar between the different categories under study (i.e. non-deformed, antero-posterior and oblique crania). This procedure was carried out as well for both the symmetric and the asymmetric component of variation. All the analyses were carried out considering alpha = 0.05.

## Results

In geometric morphometrics, hypothesis testing is based on the linear properties of the vectors obtained after applying Procrustes analysis onto the raw data (2 o 3 dimensional landmark coordinates). Later those vectors (i.e. the shape and size components of form) are used as analogs of the traditional interlandmark ("point to point") vectors. As a result, the linearity of the shape vectors allow to apply the toolkit of standard multivariate statistical analyses in order to explore (i.e. PCA), and contrast (i.e. CVA, PERMANOVA, PLS) hypotheses for assessing the pattern of observed shape variation.

### Measurement error

The Procustes ANOVA used to measure intra-observer error in the sub-sample showed that the mean square for individual variation exceeded measurement error, so the effect of measurement error was negligible (see S2 Table for further details). Measurement error was also quantified as repeatability using a ratio of the among-individual to the sum of the among-individual and measurement error components as explained in [33]. Shape repeatability was 0.955, which indicates a minimal ~4% error.

### Canonical variate analysis

**Fig 3:**
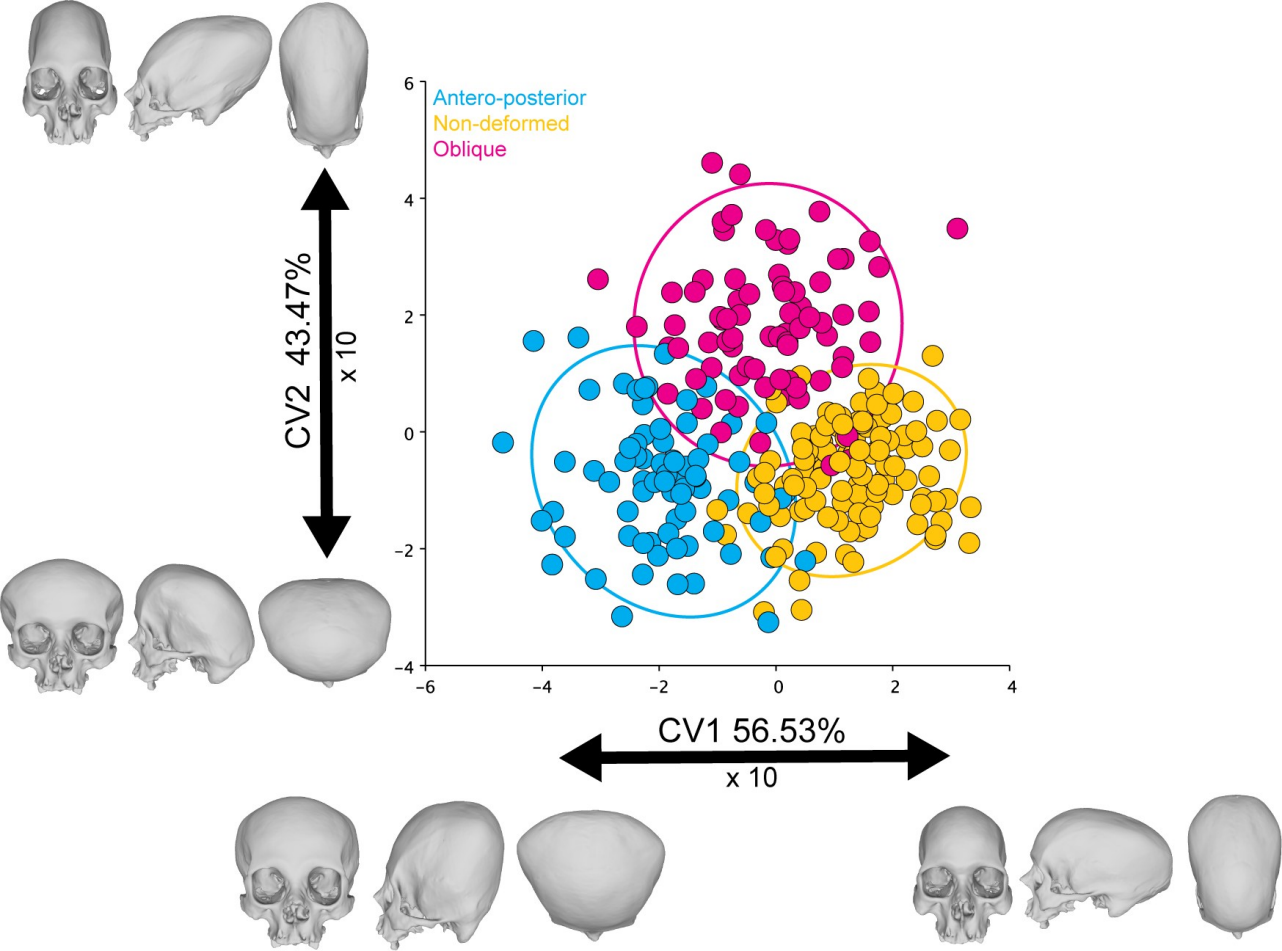
CVA of the symmetric component of the complete dataset. The ellipses represent the 90% confidence intervals.

Antero-posteriorly deformed and non-deformed skulls were significantly different (Mahalanobis distance: 3.2198; P-value: <0.0001; 10.000 perm.), as well as antero-posterior and oblique deformed crania (Mahalanobis distance: 3.2177; P-value: <0.0001; 10.000 perm.) and non-deformed and oblique skulls (Mahalanobis distance: 2.8743; P-value: <0.0001; 10.000 perm.). There was only a slight overlap between them (Fig 3) that could perhaps be attributed to either misclassification or due to the presence of skulls showing only slight deformations. The skulls presenting an antero-posterior deformation seem to be the most different ones, as observable in the higher distance between these individuals and the other cranial categories (i.e. antero-posterior, and non-deformed skulls).

### Principal component analysis

**Fig 4:**
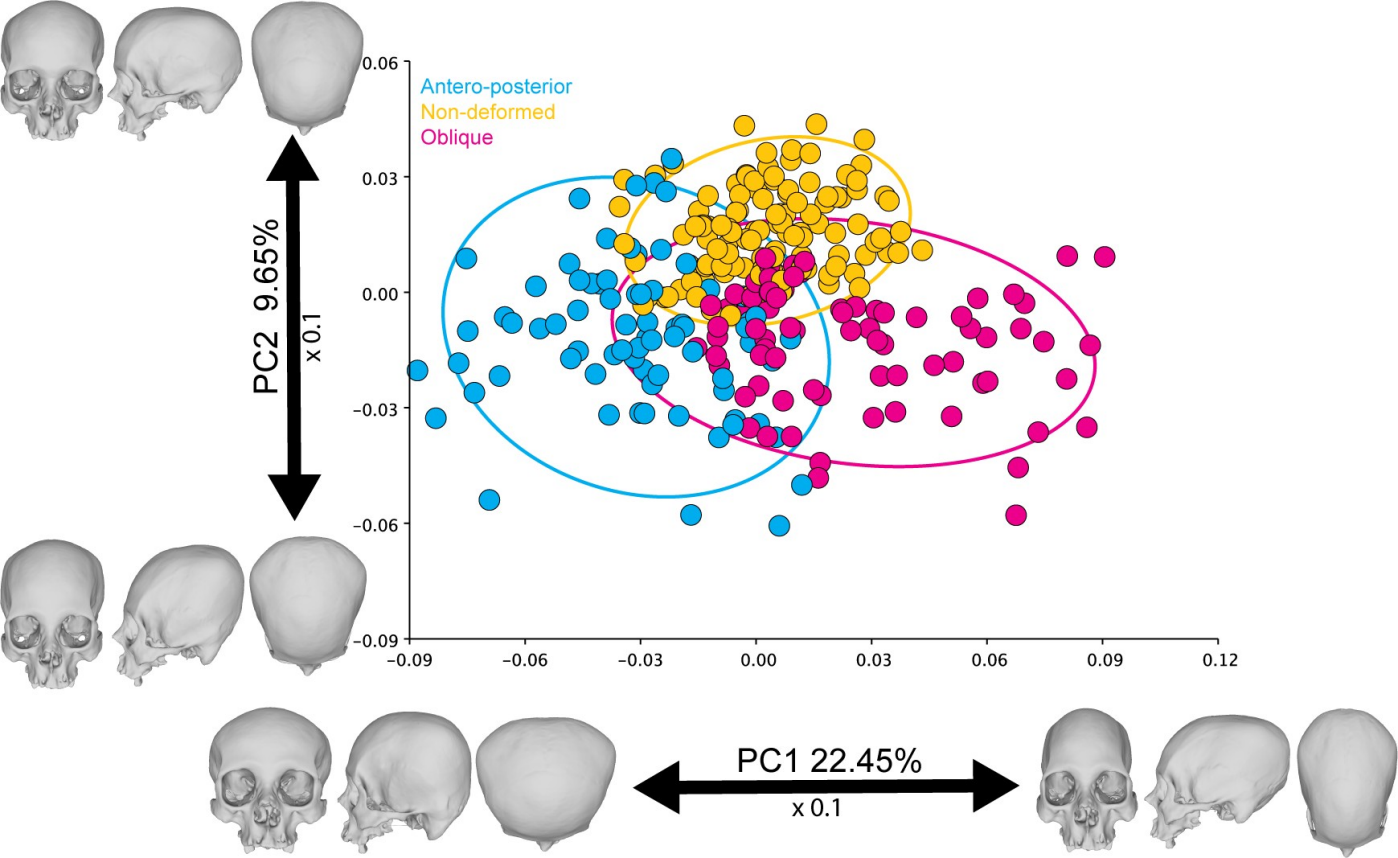
PCA of the symmetric component of the complete dataset. The ellipses represent the 90% confidence intervals.

Morphological variation was relatively widespread in the different PCs obtained from the symmetric component. The first two components accounted for 24.39% and 10.17% of the total shape variation, respectively, depicting some group separation along PC1between deformation types and non-deformed skulls (Fig 4). PC1 noticeably separated between the deforming styles. The skulls with an antero-posterior deformation were located at the left of the scatter plot, while the oblique ones were situated at the right. The non-deformed crania were positioned at the center between the two deforming styles, although separated from them by PC2. The shape changes associated with PC1 can be described as an elongation of the cranial vault towards the right of the scatter plot, while the skulls located at the left exhibit more flattened occipital and frontal bones. On the other hand, PC2 can be related to a relative elevation of the occipital landmarks and a retraction of the upper cranial vault coordinates.

### Pairwise PERMANOVAs

There were significant differences between the shape of the different cranial categories when comparing them using the PERMANOVA (Table 3). Therefore, the analyzed cranial categories are distinguishable in spite of the slight overlap observed in the PCA (Fig 4).

**Table 3:** Shape differences.

Cranial category	F-test	R^2^	Adjusted P-value (Holm-Bonferroni correction)
**Antero-posterior vs. oblique**	29.77004	0.16652702	0.003
**Antero-posterior vs. non-deformed**	26.02839	0.12048598	0.003
**Oblique vs. non-deformed**	19.4387	0.09150263	0.003

Pairwise PERMANOVA tests used to test for shape differences between cranial categories.

### Covariance ratio test

The CR tests applied to test the null hypothesis that the human skull does not show a modular behavior between viscero and neurocranium was rejected for both the symmetric (CR: 0.846; P-value: 0.007; 10.000 perm.) and the asymmetric (CR: 0.637; P-value: 0.023; 10.000 perm.) components of variation. This null hypothesis was also rejected when running the analyses separately for each one of the categories under analysis (i.e. antero-posterior, non-deformed and oblique crania) (see S1 Fig for further details).

### Partial least-squares analysis

#### Symmetric component

**Fig 5:**
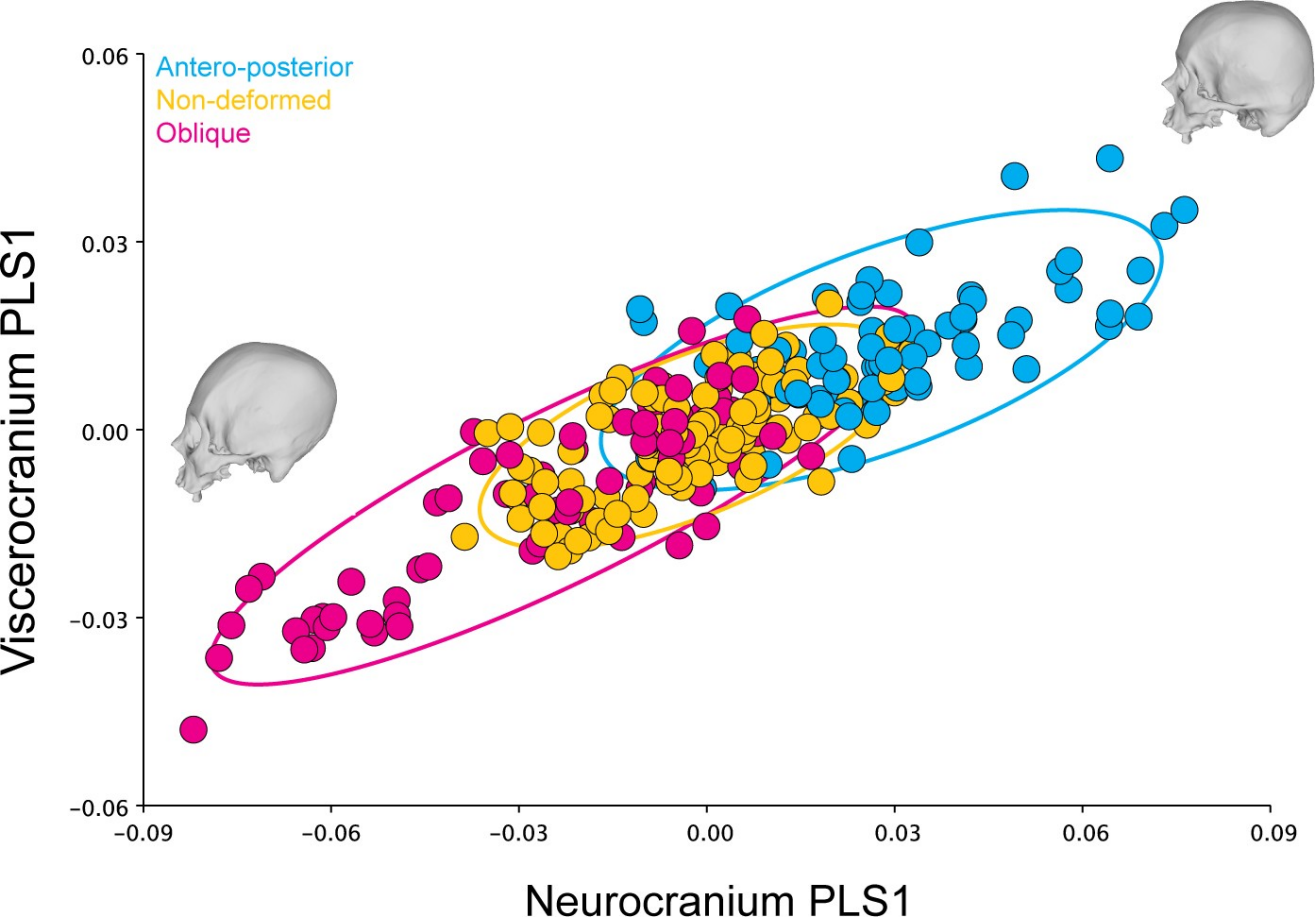
PLS1 correlation plot of the complete dataset (symmetric component) for the neurocranium and viscerocranium. The ellipses represent the 90% confidence intervals.

**Fig 6:**
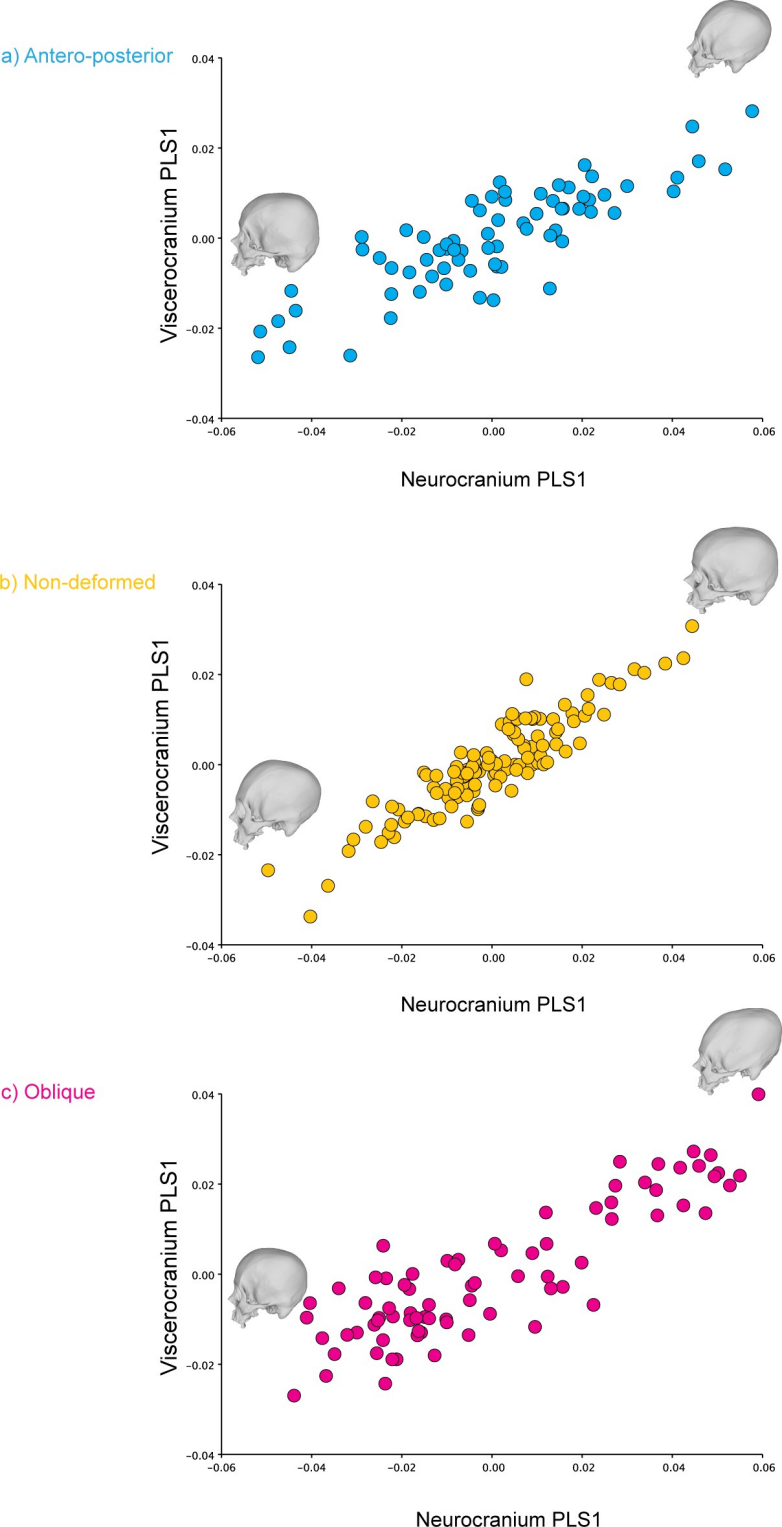
PLS1 correlation plots for the different cranial categories here analyzed (symmetric component. a) Antero-posterior; b) Non-deformed and; c) Oblique.

The analysis identified the characteristics of shape variation that most covary between the two blocks and highlighted their relative contribution to the total amount of covariation between blocks (Tables 4 and 5). The overall integration measured using the r-PLS was 0.851 (P-value: 0.0001; 10.000 perm.), whereas the PLS1 axes explained 73.55% of the total covariance of the sample (P-value: <0.0001; 10.000 perm.) (Fig 5). This percentage of explained total covariance was similar to the values obtained when analyzing each cranial category separately. The anterior-posterior (r-PLS: 0.845; P-value: 0.0001; 10.000 perm.), the oblique deformed skulls (r-PLS: 0.857; P-value: 0.0001; 10.000 perm.) and the non-deformed skulls also showed relatively similar values, although they were slightly higher in non-deformed skulls. (r-PLS: 0.888; P-value: 0.0001; 10.000 perm.). However, the antero-posterior (PLS1: 52.96% of explained covariance; P-value: <0.0001; 10.000 perm.) and the non-deformed skulls (PLS1: 51.95% of explained covariance; P-value: <0.0001; 10.000 perm.) showed a covariation pattern that was more evenly distributed in several PLS axes, as compared to the oblique crania (PLS1: 75.47% of explained covariance; P-value: <0.0001; 10.000 perm.), which showed a higher percentage of covariance explained by PLS1 (Table 6 and Fig 6). In addition, the results for PLS2 are visually presented in S2 Fig.

**Table 4:** PLS results of the complete dataset.

Type of shape component	PLS	Singular value	P-value (perm.)	% total covariance	Correlation	P-value (perm.)
**Symmetric component**	PLS1	0.00033482	<0.0001	73.55	0.85774	<0.0001
	PLS2	0.0001568	<0.0001	16.131	0.93347	<0.0001
	PLS3	0.00008937	<0.0001	5.24	0.55733	0.0018
	PLS4	0.00005396	<0.0001	1.91	0.45688	0.0006
	PLS5	0.00003998	<0.0001	1.049	0.38626	0.0027
	PLS6	0.00003208	<0.0001	0.675	0.4065	<0.0001
	PLS7	0.00002996	<0.0001	0.589	0.37694	0.0007
	PLS8	0.00001957	0.0054	0.251	0.34792	0.0018
	PLS9	0.00001893	<0.0001	0.235	0.31456	0.007
	PLS10	0.00001563	0.0002	0.16	0.34212	0.0003
	PLS11	0.00001043	0.2282	0.071	0.28316	0.0108
	PLS12	0.00000843	0.4457	0.047	0.22377	0.1982
	PLS13	0.00000753	0.2289	0.037	0.17127	0.719
	PLS14	0.00000578	0.5397	0.022	0.18892	0.2476
	PLS15	0.00000519	0.2268	0.018	0.20055	0.0414
	PLS16	0.00000437	0.1146	0.013	0.16144	0.1452
	PLS17	0.00000214	0.699	0.003	0.17011	0.0831
**Asymmetric component**	PLS1	0.00004092	< .0001	76.517	0.85205	< .0001
	PLS2	0.0000178	< .0001	14.478	0.64862	< .0001
	PLS3	0.00000822	0.0079	3.089	0.51247	< .0001
	PLS4	0.00000745	0.0001	2.537	0.28785	0.0584
	PLS5	0.0000058	0.0007	1.537	0.30112	0.0065
	PLS6	0.00000392	0.0667	0.703	0.23064	0.2107
	PLS7	0.00000316	0.0671	0.456	0.21981	0.205
	PLS8	0.00000237	0.1713	0.257	0.24704	0.0407
	PLS9	0.00000219	0.0137	0.218	0.21242	0.1552
	PLS10	0.00000144	0.3728	0.094	0.2071	0.0821
	PLS11	0.00000116	0.3329	0.061	0.1865	0.085
	PLS12	0.00000083	0.4822	0.032	0.10635	0.8422
	PLS13	0.00000069	0.0815	0.022	0.13983	0.2208

Singular values and pairwise correlations of the PLS scores between corresponding blocks (i.e. neurocranium and viscerocranium) for the complete dataset (10.000 perm.).

**Table 5:** Singular values and pairwise correlations of the PLS scores between blocks for the different cranial categories.

Cranial category	PLS	Singular value	P-value (perm.)	% total covariance	Correlation	P-value (perm.)
**Antero-posterior**	PLS1	0.00022702	<0.0001	52.957	0.84244	<0.0001
	PLS2	0.00014615	<0.0001	21.948	0.80669	<0.0001
	PLS3	0.00009995	<0.0001	10.266	0.59511	0.2154
	PLS4	0.00007074	0.005	5.142	0.50818	0.4959
	PLS5	0.00005088	0.1631	2.66	0.59985	0.0259
	PLS6	0.00004936	0.0045	2.504	0.55449	0.0557
	PLS7	0.00003657	0.1146	1.374	0.44052	0.5675
	PLS8	0.00003181	0.0728	1.039	0.51616	0.0648
	PLS9	0.00002688	0.0906	0.742	0.47659	0.147
	PLS10	0.00002189	0.2138	0.492	0.42679	0.3276
	PLS11	0.00001675	0.6639	0.288	0.42802	0.202
	PLS12	0.00001426	0.6423	0.209	0.35027	0.6087
	PLS13	0.00001172	0.713	0.141	0.3684	0.3146
	PLS14	0.00001034	0.4734	0.11	0.30987	0.5///849
	PLS15	0.00000846	0.4329	0.074	0.28601	0.5716
	PLS16	0.00000594	0.6884	0.036	0.31792	0.1609
	PLS17	0.00000413	0.6133	0.018	0.20155	0.8294
**Non-deformed**	PLS1	0.00015927	<0.0001	51.948	0.91448	<0.0001
	PLS2	0.00010278	<0.0001	21.632	0.84507	<0.0001
	PLS3	0.00007465	<0.0001	11.413	0.621	0.0335
	PLS4	0.00004542	0.0205	4.225	0.51909	0.0647
	PLS5	0.00003983	0.0042	3.249	0.52602	0.0098
	PLS6	0.00003433	0.0016	2.413	0.43808	0.1617
	PLS7	0.00003128	0.0001	2.003	0.47722	0.0111
	PLS8	0.00002324	0.0168	1.106	0.38172	0.31
	PLS9	0.00001893	0.0671	0.734	0.35914	0.3731
	PLS10	0.000014	0.5889	0.402	0.37862	0.1328
	PLS11	0.00001231	0.4508	0.311	0.32843	0.3824
	PLS12	0.00001062	0.3832	0.231	0.33221	0.2094
	PLS13	0.0000077	0.8936	0.122	0.3121	0.2286
	PLS14	0.00000725	0.5126	0.107	0.2775	0.3353
	PLS15	0.00000498	0.9274	0.051	0.17241	0.9729
	PLS16	0.00000431	0.6768	0.038	0.18569	0.7411
	PLS17	0.0000028	0.5845	0.016	0.25283	0.1212
**Oblique**	PLS1	0.00036424	<0.0001	75.467	0.87452	<0.0001
	PLS2	0.00014999	<0.0001	12.797	0.92793	<0.0001
	PLS3	0.00007936	0.0258	3.582	0.67348	0.0249
	PLS4	0.0000714	0.0009	2.9	0.57496	0.1397
	PLS5	0.00005595	0.0068	1.781	0.54332	0.1515
	PLS6	0.00004043	0.2076	0.93	0.55662	0.0409
	PLS7	0.00003977	0.0046	0.9	0.46563	0.3604
	PLS8	0.00003243	0.0112	0.598	0.50844	0.0653
	PLS9	0.00002395	0.2544	0.326	0.35908	0.8733
	PLS10	0.00002257	0.0313	0.29	0.49321	0.0333
	PLS11	0.00001596	0.5815	0.145	0.43106	0.1573
	PLS12	0.0000148	0.195	0.125	0.53239	0.0016
	PLS13	0.00001192	0.3287	0.081	0.38458	0.2015
	PLS14	0.00000834	0.8654	0.04	0.37618	0.1294
	PLS15	0.00000618	0.9523	0.022	0.28098	0.6061
	PLS16	0.00000473	0.9139	0.013	0.26312	0.4833
	PLS17	0.00000305	0.7927	0.005	0.1995	0.8789

Symmetric component.

**Table 6:** Singular values and pairwise correlations of the PLS scores between blocks for the different cranial categories: asymmetric component.

Cranial category	PLS	Singular value	P-value (perm.)	% total covariance	Correlation	P-value (perm.)
**Antero-posterior**	PLS1	0.00006503	<0.0001	74.488	0.89509	<0.0001
	PLS2	0.00003158	<0.0001	17.57	0.69941	0.0005
	PLS3	0.00001375	0.3215	3.332	0.47458	0.2284
	PLS4	0.00001025	0.45	1.85	0.48418	0.0982
	PLS5	0.00000868	0.2264	1.327	0.53345	0.0086
	PLS6	0.00000586	0.7514	0.606	0.42417	0.184
	PLS7	0.00000414	0.9481	0.303	0.35894	0.4781
	PLS8	0.00000377	0.6266	0.25	0.31224	0.7608
	PLS9	0.00000258	0.948	0.117	0.40304	0.1411
	PLS10	0.00000199	0.9701	0.07	0.2755	0.803
	PLS11	0.00000157	0.9498	0.044	0.21466	0.9477
	PLS12	0.00000145	0.5391	0.037	0.18816	0.9472
	PLS13	0.00000062	0.9791	0.007	0.24791	0.3926
**Non-deformed**	PLS1	0.00003401	<0.0001	70.459	0.83665	<0.0001
	PLS2	0.00001627	<0.0001	16.118	0.68219	<0.0001
	PLS3	0.00000932	0.1208	5.287	0.43128	0.1304
	PLS4	0.00000723	0.2256	3.18	0.42484	0.0507
	PLS5	0.00000574	0.3121	2.005	0.35483	0.2513
	PLS6	0.00000421	0.6866	1.08	0.32847	0.2929
	PLS7	0.00000375	0.3475	0.858	0.20194	0.989
	PLS8	0.00000251	0.8762	0.383	0.3035	0.2943
	PLS9	0.00000205	0.8267	0.257	0.3019	0.2034
	PLS10	0.00000173	0.6797	0.183	0.23182	0.6427
	PLS11	0.00000129	0.7887	0.102	0.19566	0.7694
	PLS12	0.00000105	0.5824	0.067	0.15091	0.9182
	PLS13	0.00000059	0.8039	0.021	0.12506	0.9397
**Oblique**	PLS1	0.00003953	<0.0001	67.032	0.85317	<0.0001
	PLS2	0.0000171	0.1233	12.547	0.58171	0.0527
	PLS3	0.00001355	0.1152	7.871	0.52199	0.0853
	PLS4	0.00001037	0.2228	4.615	0.49875	0.0577
	PLS5	0.00000889	0.0825	3.391	0.36861	0.6503
	PLS6	0.00000649	0.3315	1.807	0.35462	0.6034
	PLS7	0.00000511	0.4062	1.121	0.31515	0.7753
	PLS8	0.00000426	0.2876	0.779	0.30781	0.7411
	PLS9	0.00000266	0.9539	0.303	0.34745	0.3425
	PLS10	0.00000244	0.6727	0.256	0.2689	0.7634
	PLS11	0.00000199	0.5659	0.17	0.27819	0.4924
	PLS12	0.00000133	0.7774	0.076	0.13281	0.9981
	PLS13	0.00000087	0.6378	0.032	0.18318	0.8439

Asymmetric component

The overall integration of shape variation component measured using the r-PLS was 0.826 (P-value: 0.001; 10.000 perm.), whilst the PLS1 axes explained 76.52% of the total covariance of the sample (P-value: <0.001; 10.000 perm.). This value was relatively similar to the ones obtained for the antero-posterior (r-PLS: 0.879; P-value: 0.001; 10.000 perm.) and oblique (r-PLS: 0.868; P-value:0.001; 10.000 perm.) crania, while the non-deformed skulls showed a lower integration value (r-PLS: 0.735; P-value:0.001; 10.000 perm.). Both oblique (PLS1: 67.03% of explained covariance; P-value: <0.001; 10.000 perm.) and non-deformed skulls (PLS1: 70.46% of explained covariance; P-value: <0.001; 10.000 perm.) exhibited a similar distribution of explained covariance by their PLS axes. However, the antero-posterior deformed crania a higher level of integration in their first PLS axes (PLS1: 74.49% of explained covariance; P-value: <0.001; 10.000 perm.) (Table 6).

### Angular comparison between PLS axes

#### Symmetric component

From all the possible comparisons between the paired singular axes between non-deformed skulls and the antero-posterior and oblique crania, only a few of them were significant. Below are presented the comparisons that were significant for the two PLS axes that accounted for the majority of the total covariance for the symmetric component.

Antero-posterior PLS2 v/s non-deformed PLS2 (θ = 62.762°; P-value = 0.00094)

Antero-posterior PLS1 v/s oblique PLS1 (θ = 61.069°; P-value = 0.00043)

Antero-posterior PLS1 v/s oblique PLS2 (θ = 64.017°; P-value = 0.00164)

Non-deformed PLS1 v/s oblique PLS2 (θ = 14.579°; P-value <0.00001)

Non-deformed PLS2 v/s oblique PLS1 (θ = 41.117°; P-value <0.00001)

#### Asymmetric component

From all the possible comparisons between the paired singular axes between non-deformed skulls and the antero-posterior deformed and oblique crania, just few of them were significant. Below are presented the comparisons that were significant for the two PLS axes that accounted for the majority of the total covariance for the asymmetric component.

Antero-posterior PLS1 v/s non-deformed PLS1 (θ = 39.064°; P-value <0.00001)

Antero-posterior PLS2 v/s non-deformed PLS2 (θ = 42.940°; P-value <0.00001)

Antero-posterior PLS1 v/s oblique PLS1 (θ = 28.325°; P-value <0.00001)

Non-deformed PLS1 v/s oblique PLS1 (θ = 30.414°; P-value <0.00001)

### Angular comparison between principal components and PLS axes

The PC’s and PLS axes of the complete dataset were compared using the same procedure outlined above in order to test whether the observed pattern of variation (PC’s) followed a similar trend as the observed patterns of covariation between viscero- and neurocranium (PLS axes). In a similar fashion, from all the possible comparisons only a few of them were significant (Symmetric component: θ PC1 v/s PLS1 = 13.361°, P-value <0.00001; Asymmetric component: θ PC1 v/s PLS1 = 34.190°, P-value <0.00001; θ PC2 v/s PLS2 = 49.545°, P-value <0.00001).

### Matrix correlations

The matrix correlation between the covariance matrices for the symmetric and asymmetric components of variation for the complete dataset was 0.638 (P-value: < 0.0001; 10.000 perm.). Below are presented the pairwise matrix correlation results for the different cranial categories for both the symmetric and asymmetric component of variation.

#### Symmetric component

Antero-posterior v/s non-deformed: 0.618 (P-value: < 0.0001; 10.000 perm.).

Antero-posterior v/s oblique: 0.592 (P-value: < 0.0001; 10.000 perm.).

Non-deformed v/s oblique: 0.750 (P-value: < 0.0001; 10.000 perm.).

#### Asymmetric component

Antero-posterior v/s non-deformed: 0.754 (P-value: < 0.0001; 10.000 perm.).

Antero-posterior v/s oblique: 0.745 (P-value: < 0.0001; 10.000 perm.).

Non-deformed v/s oblique: 0.873 (P-value: < 0.0001; 10.000 perm.).

## Discussion

The PCA showed that even though there is morphological continuum ranging from one deforming type to the other (with non-deformed individuals relatively in between), it is possible to notice differences between the analyzed cranial categories. This was confirmed when running the pairwise PERMANOVAs and CVA analysis since highly significant differences between the two deforming styles and the non-deformed crania were observed. There was a slight overlap between the groups that was expected and coherent with the morphological continuum outlined above. It is interesting that in spite of the several proposed classifications to define deforming styles [72,93,111–115], the simple binomial sorting applied here followed by a numerical shape analysis, allowed a well-defined distinction between dissimilar deforming traditions. This result has taxonomic implications related to the a priori nature and the qualitative character of the classifications usually applied in bioanthropoloy and archaeology to give diagnostics about specimens’ provenance and grouping [116]. An almost inevitable consequence of this typological approach is the over-representation of categories and subcategories defining each group of objects [117]. Future studies should test whether more complex classification schemes have an enhanced classificatory power when compared to the simplified grouping used in this research. In addition, it is interesting that the two deformed cranial categories display more variation along the two first PCs as compared to the non-deformed crania. Although these differences in variation are not completely unexpected, they represent a good example how developmental plasticity acts on the human skull. Environmental perturbations such as the ACD influence growth and development. Hence, the application of these deforming devices during early years of post-natal life is likely related to an increase in morphological variance in the two deformed groups, explained in turn, as has been observed by several authors, by the increase of the vertical development of the maxilla [118], by changes in basicranial shape and in cranial base angles, as well as in the relative position of the mandibular fossae [92], significantly greater frequencies of lambdoid ossicles, and more lambdoid wormian bones [119], and a significant greater level of bilateral asymmetry [120,121], compared to the non-deformed group.

The null hypothesis stating that there was no modular behavior on the skull based on two different developmental origins was rejected. For the symmetric component of variation, the CR coefficient was significantly lower than one, thus suggesting that there was a degree of independence between the two modules. The asymmetric component also showed a lower and significant CR value, which suggest a strong degree of independence between the two modules. Thus, there is support for the hypothesis that the human cranium displays significant modularity when compared to the null hypothesis of no modular structure. It is relevant to keep in mind that modularity and integration are not two ends of the same continuum, thus it is perfectly possible to have both modularity and integration in the same dataset as in the present case. Modularity implies greater covariation of variables within modules than between them, whereas integration means that two modules are more correlated than it would be expected from chance by random arrangements of pairs of observations from each module. Hence, it is possible to have both integration between modules and modularity within them.

Regarding the static morphological integration of the human cranium, it seems that the different categories analyzed here show relatively similar overall levels of integration as observed in their r-PLS values. Nevertheless, the oblique deforming style appears to constrain the strength of static covariation between the viscero and the neurocranium in a more defined direction as compared with both the antero-posterior crania and the non-deformed skulls (Table 4). Oblique skulls exhibit a covariance greater than observed on the antero-posterior and the non-deformed individuals for PLS1. This could mean that the normal growing vectors of the skull are at least partially canalized towards the inner portion of the facial skeleton due to the application of the deforming device, consequently increasing the covariation with the calvarium. Interestingly the PLS1 for the oblique skulls (symmetric component) accounted for 75.467% (P-value: <0.0001) of the covariance of the sample, while the PLS1 for non-deformed skulls accounted for only the 51.948% (P-value: <0.0001) and 52.957% (P-value: <0.0001) for the antero-posterior deformed crania. These latter categories exhibit a covariation pattern that is more distributed in other directions (i.e. axes) as compared with oblique skulls. One measure of the morphological integration level is the correlation between the first PLS scores of the analyzed blocks, which was higher for the oblique skulls as compared to the other crania (Table 4) [20,122]. It is evident from these results that the oblique deforming style produces a more directed static covariation pattern between the viscero and neurocranium (Fig 6C). The skulls at the top of the correlation plot have more elongated and oblique neurocrania associated to more prognathic faces, while the skulls at the bottom have more rounded cranial vaults. These results are in agreement with previous studies that have found that the ACD effect is not confined only to the braincase but also affects the skeletal face [81,83]. On the other hand, the correlation plot of the PLS1 for non-deformed skulls showed that crania at the top of the plot exhibited more flattened neurocrania, while at the bottom the skulls showed a more rounded cranial vault. In the case of the antero-posterior deformed skulls, those individuals with more flattened occipitals were at the bottom of the plot, while those at the top showed more inclined cranial vaults. PLS2 also exhibits some interesting patterns (S2 Fig). Whereas the PLS1 shows, in a similar fashion as the PCA, a morphological continuum ranging from one deforming type to the other (with non-deformed individuals in between) (Fig 5), the PLS2 correlation plot of the complete dataset (symmetric component) shows a total overlap between the categories (S4A Fig). From morphological perspective, the crania at the top of this correlation plot display more elongated and laterally expanded neurocrania with a depressed area between the frontal and the parietals, whilst the skulls at the bottom show more rounded and vertically expanded vaults and flatter faces. PLS2 correlation plots for each one the cranial categories (symmetric component) can be found in S2B, S2C and S2D Fig.

Conversely, the developmental integration analysis showed that non-deformed skulls exhibit an overall lower level of integration as compared to the deformed skulls as shown by their r-PLS values. This might mean that the deforming styles increase the covariation between the asymmetric components of the neuro and viscerocranium. This means that an increased asymmetry in one of these cranial parts would generate a stronger increase of the asymmetry of the other skull compartment as compared to the non-deformed skulls, or a decreased asymmetry in one block would produce a stronger decrease of the asymmetry of the other cranial portion. In addition, the antero-posterior deformed crania showed a higher level of developmental integration in their first PLS axes (PLS1: 74.488% of explained covariance; P-value: <0.001; 1,000 perm.), which suggests that this particular deforming style directs the covariation between the two cranial blocks in a more directed way (i.e. it is less distributed in other directions of covariation).

ACD affects the morphological integration of the skull, showing a particular covariation trend depending on the deforming style [92]. It seems that the different deforming types produce different covariation patterns between the viscero and neurocranium as observed by the total covariance explained by each PLS axes for each one of the shape variation components under analysis. One plausible explanation is that the different deforming devices exerted different force vectors (both in direction and magnitude) on the neurocranium. These differences in force vectors were generated probably by the different materials of the deforming devices (i.e. wood, fibers, ropes, pads combined with flatted stones, etc.) and by the exact anatomical location where the deforming device was placed.

Another perhaps more important explanation to the different degree of integration between modules in deformed and non-deformed crania, is the change in the normal loads acting on cranial bones during ACD. There is an important role of mechanical loads in bone growth and development [123–128]. In the human cranium, the most noticeable source of loads is mastication, and its effect has been shown to be more important in the lower face [16]. The remaining cranial structures including the neurocranium reflect other non-load-bearing aspects such as population history and climate [129,130] in a stronger manner compared to the mandible [131,132]. ACD could act by affecting this modular pattern of load distribution, increasing the strength of the covariation between the neurocranium and the face in a more constrained direction by re-distributing intracranial pressure during development. This new, altered pattern of load likely impacts on a developing face that is now not only under the effect of the mastication and the functional matrices, thus allowing the deformation to reflect at a facial level.

When quantitatively testing if the observed covariation patterns were similar or not depending on the analyzed cranial category, the angular comparison between the PLS vectors shown that the majority of them were dissimilar (i.e. their angular difference was near 90°). However, there were some significant similarities between some PLS axes that could be related to the fact that despite the differences in the intensity of the covariation, the overall covariation pattern could be relatively similar. The angular comparison analyses also showed that there was a significant relationship between the PC’s and the PLS axes, thus indicating that the patterns of observed variation are in concordance with the observed covariation patterns between viscero and neurocranium. This could mean that the observed variation in cranial shape is due to the morphological integration of the skeletal face and cranial vault.

Finally, the matrix correlation analysis between the symmetric and asymmetric components of variation showed a moderate-to-high value. When carried out the matrix correlations by separating the different cranial categories it was observed that for both the symmetric and asymmetric components of variation, non-deformed skulls are more similar to the oblique crania. This seems to indicate that in spite of the increased static covariation observed in PLS1 of the oblique crania, they are more similar to the non-deformed condition. These results are concordant with the CVA results that showed that antero-posterior deformed skulls are the most different of the three cranial categories.

## Conclusions

The results from this research show that there is a modular organization of the human skull (i.e. neuro and viscerocranium). Furthermore, the present results show that the strength of the morphological integration between the neurocranium and viscerocranium is differentially augmented depending on the applied force vectors on the skull (i.e. oblique deforming style). Compressive forces onto the parietal bones (i.e. oblique ACD) increases the static morphological integration between these two anatomical regions, while compressive forces onto the occipital and frontal bones (i.e. antero-posterior ACD), increases the developmental integration of the skull. Although the underlying cause of this phenomenon is still unknown, it could be related with the specific mechanisms constraining the normal expansion of the brain and how this affects the normal growth and development of the skull. Further analyses are required to get a better insight of the possible effects of ACD on human biology. One interesting approach would be to use the present results to carefully design a biomechanical simulation of the growing skull while simulating compressive forces as proxies for the different deforming devices.

## Supporting information

S1 Table Sample used in this study. Individual numbers and complete repository information, including museum name and geographic location.
(CSV)

S2 Table Intra-observer error Procrustes ANOVA. Mean squares values, degrees of freedom (df), F statistic, and statistical significance (P value) of intra-observer error.
(CSV)

S3 Table Posterior probabilities from the cross-validated CVA. Linear Discriminant Analysis using the Procrustes coordinates of the first 11 PCs after a broken-stick model for 3 classes /'Antero-Posterior', 'Non.deformed', 'Oblique'/.
(CSV)

S1 Fig CR coefficients. CR coefficients obtained from permutation tests (999 rounds) of alternative partitions of a) the complete dataset, b) antero-posterior deformed skulls, c) non-deformed crania and d) the oblique sample, with the observed CR coefficients designated by a red arrow. Both the symmetric and asymmetric components of shape variation were analyzed.
(PDF)

S2 Fig PLS2 correlation plots (symmetric component). a) the Complete dataset, as welll as for the different cranial categories under analysis b) Antero-posterior; c) Non-deformed and; d) Oblique.
(PDF)

## References

[1] Dembo A, Imbelloni J. Deformaciones intencionales del cuerpo humano de carácter étnico. Editori Nova; 1938. 366 p.

[2] Moss M. The functional matrix In: Kraus B, Riedel R, editors. Vistas in orthodontics. Philadelphia: Lea & Febiger; 1962 p. 85–98.

[3] Lieberman DE. Evolution of the human head. 1 edition Cambridge: Harvard University Press; 2011. 728 p.

[4] Klingenberg CP. Cranial integration and modularity: insights into evolution and development from morphometric data. Hystrix Ital J Mammal. 2013;24(1):43–58.

[5] Depew MJ, Sharpe PT. Craniofacial development In: Rossant, Tam PPL, editors. Mouse development: patterning, morphogenesis, and organogenesis. San Diego: Academic Press; 2002 p. 421–498.

[6] Lieberman DE. Speculations about the selective basis for modern human craniofacial form. Evol Anthropol Issues News Rev. 2008 Jan;17(1):55–68.

[7] Klingenberg CP. Morphological integration and developmental modularity. Annu Rev Ecol Evol Syst. 2008;39(1):115–32.

[8] Cheverud JM. Phenotypic, genetic, and environmental morphological integration in the cranium. Evolution. 1982 5 1;36(3):499–516. 10.1111/j.1558-5646.1982.tb05070.x 28568050

[9] Klingenberg CP. Studying morphological integration and modularity at multiple levels: concepts and analysis. Philos Trans R Soc Lond B Biol Sci. 2014 8 19;369(1649):20130249 10.1098/rstb.2013.0249 25002695 PMC4084535

[10] von Cramon-Taubadel N. Multivariate morphometrics, quantitative genetics, and neutral theory: Developing a “modernsynthesis” for primate evolutionary morphology. Evol Anthropol. 2018;28:21–33. 10.1002/evan.21761 30652384

[11] Adams D, Felice R. Assessing trait covariation and morphological integration on phylogenies using evolutionary covariance matrice. PLOS One. 2014;9:e94335 10.1371/journal.pone.0094335 24728003 PMC3984176

[12] van der Klaauw CJ. Size and position of the functional components of the skull. Arch Néerl Zool. 1948;9:1–159.

[13] Roth VL. Cranial integration in the sciuridae. Am Zool. 1996 2;36(1):14–23.

[14] Hallgrímsson B, Willmore K, Dorval C, Cooper DML. Craniofacial variability and modularity in macaques and mice. J Exp Zoolog B Mol Dev Evol. 2004;302B(3):207–225. 10.1002/jez.b.21002 15211683

[15] Bastir M, Rosas A. Hierarchical nature of morphological integration and modularity in the human posterior face. Am J Phys Anthropol. 2005;128(1):26–34. 10.1002/ajpa.20191 15778978

[16] von Cramon-Taubadel N. The relative efficacy of functional and developmental cranial modules for reconstructing global human population history. Am J Phys Anthropol. 2011 9;146(1):83–93. 10.1002/ajpa.21550 21710659

[17] Klingenberg CP. Morphological integration and developmental modularity. Annu Rev Ecol Evol Syst. 2008;39(1):115–132.

[18] Cheverud JM. Morphological integration in the Saddle-Back Tamarin (Saguinus fuscicollis) Cranium. Am Nat—AMER Nat. 1995;145(1).

[19] Lieberman DE, McBratney BM, Krovitz G. The evolution and development of cranial form in Homo sapiens. Proc Natl Acad Sci. 2002 5 2;99(3):1134–9. 10.1073/pnas.022440799 11805284 PMC122156

[20] Bookstein FL, Gunz P, Mitterœcker P, Prossinger H, Schæfer K, Seidler H. Cranial integration in Homo: singular warps analysis of the midsagittal plane in ontogeny and evolution. J Hum Evol. 2003 2;44(2):167–87. 10.1016/s0047-2484(02)00201-4 12662941

[21] Ackermann RR. Ontogenetic integration of the hominoid face. J Hum Evol. 2005 2;48(2):175–97. 10.1016/j.jhevol.2004.11.001 15701530

[22] Goswami A. Cranial modularity shifts during mammalian evolution. Am Nat. 2006 8 1;168(2):270–80. 10.1086/505758 16874636

[23] Goswami A, Janis C. Morphological integration in the carnivoran skull. Evolution. 2006 1 1;60(1):169–83. 16568641

[24] Mitteroecker P, Bookstein F. The evolutionary role of modularity and integration in the Hominoid cranium. Evolution. 2008 4 1;62(4):943–58. 10.1111/j.1558-5646.2008.00321.x 18194472

[25] Püschel T. Modularidad e integración morfológica en cráneos humanos: un enfoque morfométrico geométrico. Int J Morphol. 2014 3;32(1):299–304.

[26] Kawasaki K, Richtsmeier J. Association of the chondrocranium and dermatocranium in early skull development Percival, CJ and JT Richtsmeier (Eds.), Building Bones: Bone Development and Formation in Anthropology, Cambridge Studies in Biological and Evolutionary Anthropology. Cambridge University Press Pp 52–78.

[27] Polanski JM, Franciscus RG. Patterns of craniofacial integration in extant Homo, Pan, and Gorilla. Am J Phys Anthropol. 2006 9;131(1):38–49. 10.1002/ajpa.20421 16552733

[28] Bruner E. Comparing endocranial form and shape differences in modern humans and Neandertal: a geometric approach. PaleoAnthropology. 2008;2008:93–106.

[29] Bruner E, Ripani M. A quantitative and descriptive approach to morphological variation of the endocranial base in modern humans. Am J Phys Anthropol. 2008 9;137(1):30–40. 10.1002/ajpa.20837 18398846

[30] Singh N, Harvati K, Hublin J-J, Klingenberg CP. Morphological evolution through integration: A quantitative study of cranial integration in Homo, Pan, Gorilla and Pongo. J Hum Evol. 2012 1;62(1):155–64. 10.1016/j.jhevol.2011.11.006 22178399

[31] Esteve-Altava B, Marugán-Lobón J, Botella H, Bastir M, Rasskin-Gutman D. Grist for Riedl’s mill: A network model perspective on the integration and modularity of the human skull. J Exp Zoolog B Mol Dev Evol. 2013 Dec 1;320(8):489–500. 10.1002/jez.b.22524 23913546

[32] Sperber GH, Guttman GD, Sperber SM. Craniofacial Development (Book for Windows & Macintosh). PMPH-USA; 2001. 236 p.

[33] Zelditch ML, Swiderski DL, Sheets HD. General linear} models In: Geometric morphometrics for biologists. 2 edition. San Diego: Academic Press; 2012 p. 209–228.

[34] Lieberman DE, Ross CF, Ravosa MJ. The primate cranial base: ontogeny, function, and integration. Am J Phys Anthropol. 2000;Suppl 31:117–169. 10.1002/1096-8644(2000)43:31+<117::aid-ajpa5>3.3.co;2-9 11123839

[35] Profico A, Piras P, Buzi C, Di Vincenzo F, Lattarini F, Melchionna M, et al The evolution of cranial base and face in Cercopithecoidea and Hominoidea: Modularity and morphological integration. Am J Primatol. 2017 12;79(12):e22721. 10.1002/ajp.22721 29095513

[36] Neaux D, Sansalone G, Ledogar JA, Heins Ledogar S, Luk THY, Wroe S. Basicranium and face: Assessing the impact of morphological integration on primate evolution. J Hum Evol. 2018 5;118:43–55. 10.1016/j.jhevol.2018.02.007 29606202

[37] Neaux D, Wroe S, Ledogar JA, Heins Ledogar S, Sansalone G. Morphological integration affects the evolution of midline cranial base, lateral basicranium, and face across primates. Am J Phys Anthropol. 2019 9;170(1):37–47. 10.1002/ajpa.23899 31290149

[38] Carlson BM. Human embryology and developmental biology. Elsevier Health Sciences; 2013. 523 p.

[39] Marroig G, Cheverud JM. A comparison of phenotypic variation and covariation patterns and the role of phylogeny, ecology, and ontogeny during cranial evolution of new world monkeys. Evol Int J Org Evol. 2001 12;55(12):2576–2600. 10.1111/j.0014-3820.2001.tb00770.x 11831671

[40] Ackermann RR. Ontogenetic integration of the hominoid face. J Hum Evol. 2005 2;48(2):175–197. 10.1016/j.jhevol.2004.11.001 15701530

[41] Hallgrímsson B, Lieberman D, Liu W, Ford-Hutchinson A, Jirik F. Epigenetic interactions and the structure of phenotypic variation in the cranium. Evol Dev. 2007;9(1):76–91. 10.1111/j.1525-142X.2006.00139.x 17227368

[42] Weber J, Nauck C, Creutz U, Al-Zain F, Pusch CM. Fronto-ethmoidal encephalozele in a historical skull with artificial deformation and no signs of chronic elevated intracranial pressure. Acta Neurochir (Wien). 2008;150(10):1107–1109. 18773138 10.1007/s00701-008-0025-3

[43] Lieberman DE. Evolution of the human head 1 edition. Cambridge, Mass: Harvard University Press; 2011. 728 p.

[44] Vora SR. Mouse models for the study of cranial base growth and anomalies. Orthod Craniofac Res. 2017;20:18–25. 10.1111/ocr.12180 28643912

[45] Richtsmeier J, DeLeon V. Morphological integration of the skull in craniofacial anomalies. Orthod Craniofac Res. 2009 Aug 1;12(3):149–58. 10.1111/j.1601-6343.2009.01448.x 19627516 PMC2804975

[46] Heuzé Y, Martínez-Abadías N, Stella JM, Arnaud E, Collet C, García Fructuoso G, et al Quantification of facial skeletal shape variation in fibroblast growth factor receptor-related craniosynostosis syndromes. Birt Defects Res A Clin Mol Teratol. 2014 Apr 1;100(4):250–9. 10.1002/bdra.23228 PMC4029055 24578066

[47] Manríquez G, González-Bergás FE, Salinas JC, Espoueys O. Deformación intencional del cráneo en poblaciones arqueológicas de Arica, Chile: análisis preliminar de morfometría geométrica con uso de radiografías craneofaciales. Chungará. 2006 Jun;38(1):13–34.

[48] Dingwall EJ. Artificial Cranial Deformation: A contribution to the study of ethnic mutilations. Bale; 1931. 313 p.

[49] Gerszten PC, Gerszten E. Intentional cranial deformation: a disappearing form of self-mutilation. Neurosurgery. 1995 Sep;37(3):374–381; discussion 381–382. 10.1227/00006123-199509000-00002 7501099

[50] Bucchi A, Püschel T, Manríquez G. Artificial cranial modification in San Pedro de Atacama and the Loa basin: a quantitative approach to its role as a marker of social identity. Rev Chil Antropol. 2016;34(2):19–30.

[51] Weiss P. Osteología Cultural, Prácticas Cefálicas: 2da Parte, Tipología de las deformaciones cefálicas–Estudio cultural de los tipos cefálicos y de algunas enfermedades oseas. Lima: Universidad Nacional Mayor de San Marcos; 1961. 140 p.

[52] Fitzsimmons E, Prost J, Peniston S. Infant head molding; a cultural practice. Arch Fam Med. 1998;7:88–90. 10.1001/archfami.7.1.88 9443706

[53] Gump W. Modern induced skull deformity in adults. Neurosurg Focus. 2010;29:e4. 10.3171/2010.10.FOCUS10203 21121718

[54] Kohler G. Die künstlichedeformation des Schädels [PhD Thesis]. Friedrich-Alexanders-Universität Nürnberg; 1901.

[55] Schijman E. Artificial cranial deformation in newborns in the pre-Columbian Andes. Childs Nerv Syst. 2005;21(11):945–950. 10.1007/s00381-004-1127-8 15711831

[56] Prestigiacomo CJ, Krieger M. Deformations and malformations: the history of induced and congenital skull deformity. Neurosurg Focus. 2010;29(6):1. 21121723 10.3171/2010.12.FOCUS.Intro

[57] Mednikova M. The practice of cultural deformation of the head in its eurasian context. Opus Interdiscip Res Archaeol. 2006 5;206–229.

[58] Torres-Rouff C, Yablonsky LT. Cranial vault modification as a cultural artifact: A comparison of the Eurasian steppes and the Andes. HOMO- J Comp Hum Biol. 2005;56(1):1–16. 10.1016/j.jchb.2004.09.001 15901115

[59] Shvedchikova Ty. To the question of dissemination of artificial cranial deformation among the ancient population in Aral region. Bull Mosc Univ. 2009;23(Anthropology 1 /in Russian/):78–84.

[60] Arensburg B, Hershkovitz I. Cranial deformation and trephination in the Middle East. Bull Mém Soc Anthropol Paris. 1988;5(14–3):139–150.

[61] Özbek M. Cranial deformation in a subadult sample from Değirmentepe (Chalcolithic, Turkey). Am J Phys Anthropol. 2001;238–244. 10.1002/ajpa.1078 11424075

[62] Fletcher A, Pearson J, Ambers J. The manipulation of social and physical identity in the Pre-Pottery Neolithic. Camb Archaeol J. 2008;18(03):309.

[63] Pap I. Data to the problem of artificial cranial deformation. Ann Hist-Nat Musei Natl Hung. 1985;77:281–289.

[64] Arnold W, Fedorischeva V, Naumova E, Yabluchansky N. Craniometric measurement of artificial cranial deformations in Eastern European skulls. J Biol Clin Anthropol Stuttg. 2008;2:1–8. 18712154

[65] Enchev Y, Nedelkov G, Atanassova-Timeva N, Jordanov J. Paleoneurosurgical aspects of Proto-Bulgarian artificial skull deformations. Neurosurg Focus. 2010;29(6):E3 10.3171/2010.9.FOCUS10193 21121717

[66] Molnár M, János I, Szűcs L, Szathmáry L. Artificially deformed crania from the Hun-Germanic Period (5th–6th century ad) in northeastern Hungary: historical and morphological analysis. Neurosurg Focus. 2014;36(4):E1 10.3171/2014.1.FOCUS13466 24684322

[67] Mayall P, Pilbrow V, Bitadze L. Migrating Huns and modified heads: Eigenshape analysis comparing intentionally modified crania from Hungary and Georgia in the migration period of Europe. PLoS ONE. 2017;12(2). 10.1371/journal.pone.0171064 PMC5289542 28152046

[68] Gosse L-A. Essai sur les déformations artificielles du crâne. Libraire d. Paris: J.-B. Baillière; 1855. 170 p.

[69] Broca P. Sur la déformation toulousaine du crâne. Bull Société Anthropol Paris. 1871;2(6):100–131.

[70] Delisle F. Les deformations artificielles ud crane en France. Carte de leur distribution. Bull Memoires Soc Anthropol Paris. 1902;5(3):111–167.

[71] Tritsaroli P. Artificial cranial modification on a female skeleton from the Byzantine site of Maroneia (Thrace, Greece). Int J Osteoarchaeol. 2011;21(4):464–478.

[72] Morton SG. Crania Americana: or a comparatif view of the skulls of various aboriginal nations of … America. J. Dobson; 1839. 488 p.

[73] Munizaga J. Intentional cranial deformation in the preColumbian populations of ecuador. Am J Phys Anthropol. 1976;45(3):687–694.

[74] Perez SI. Artificial cranial deformation in South America: a geometric morphometrics approximation. J Archaeol Sci. 2007 Oct;34(10):1649–58.

[75] Morton S. Crania Americana: or a comparatif view of the skulls of various aboriginal nations of America. London: Simpkin, Marshall & Co; 1839. 488 p.

[76] Soto-Heim P. Evolución de deformaciones intencionales, tocados y practicas funerarias en la prehistoria de Arica, Chile. Chungara. 1987;(19):129–213.

[77] Torres‐Rouff C. Cranial vault modification and ethnicity in Middle Horizon San Pedro de Atacama, Chile. Curr Anthropol. 2002;43(1):163–171.

[78] Blom D. Embodying borders: Human body modification and diversity in Tiwanaku society. J Anthropol Archaeol. 2005;24(1):1–24.

[79] Torres-Rouff C. La deformación craneana en San Pedro de Atacama. Estud Atacameños. 2007;33:25–38.

[80] Cocilovo J, Varela H. La distribución de la deformación artificial del cráneo en el área andina centro sur. Relac Soc Argent Antropol. 2010;35:41–68.

[81] Anton SC. Intentional cranial vault deformation and induced changes of the cranial base and face. Am J Phys Anthropol. 1989 6;79(2):253–67. 10.1002/ajpa.1330790213 2662783

[82] Kohn LAP, Leigh SR, Jacobs SC, Cheverud JM. Effects of annular cranial vault modification on the cranial base and face. Am J Phys Anthropol. 1993 2 1;90(2):147–68. 10.1002/ajpa.1330900203 8430751

[83] Friess M, Baylac M. Exploring artificial cranial deformation using elliptic Fourier analysis of Procrustes aligned outlines. Am J Phys Anthropol. 2003 9;122(1):11–22. 10.1002/ajpa.10286 12923900

[84] Baylac M, Frieß M. Fourier descriptors, procrustes superimposition, and data dimensionality: an example of cranial shape analysis in modern human populations In: Slice DE, editor. Modern morphometrics in physical anthropology. Springer US; 2005 p. 145–65. (Developments in Primatology: Progress and Prospects).

[85] Martínez-Abadías N, Paschetta C, Azevedo S de, Esparza M, González-José R. Developmental and genetic constraints on neurocranial globularity: insights from analyses of deformed skulls and quantitative genetics. Evol Biol. 2009 2 4;36(1):37–56.

[86] Mendonça de Souza SMF, Reinhard KJ, Lessa A. Cranial deformation as the cause of death for a child from the Chillon River Valley, Peru. Chungará Arica. 2009;40(1):41–54.

[87] Anton SC, Jaslow CR, Swartz SM. Sutural complexity in artificially deformed human (Homo sapiens) crania. J Morphol. 1992 12 1;214(3):321–32. 10.1002/jmor.1052140307 1474599

[88] Cheverud JM, Kohn LA, Konigsberg LW, Leigh SR. Effects of fronto-occipital artificial cranial vault modification on the cranial base and face. Am J Phys Anthropol. 1992 7;88(3):323–45. 10.1002/ajpa.1330880307 1642320

[89] Ketoff S, Girinon F, Schlager S, Friess M, Schouman T, Rouch P, et al Zygomatic bone shape in intentional cranial deformations: a model for the study of the interactions between skull growth and facial morphology. J Anat. 2016; 10.1111/joa.12581 PMC5345617 28032345

[90] Khonsari R, Friess M, Nysjö J, Odri G, Malmberg F, Nyström I, et al Shape and volume of craniofacial cavities in intentional skull deformations. Am J Phys Anthropol. 2013;151:110–119. 10.1002/ajpa.22263 23553676

[91] Sandy R, Hennocq Q, Nysjö J, Giran G, Friess M, Khonsari RH. Orbital shape in intentional skull deformations and adult sagittal craniosynostoses. J Anat. 2018;233(3):302–310. 10.1111/joa.12844 PMC6081507 29926913

[92] Cottin M, Khonsari R, Friess M. Assessing cranial plasticity in humans: The impact of artificial deformation on masticatory and basicranial structures. Comptes Rendus Palevol. 2017;16(5–6):545–556.

[93] Dembo A, Imbelloni J. Deformaciones intencionales del cuerpo humano de carácter étnico. Editori Nova; 1938. 366 p.

[94] O’Higgins P. The study of morphological variation in the hominid fossil record: biology, landmarks and geometry. J Anat. 2000 7;197(Pt 1):103–20. 10999273 10.1046/j.1469-7580.2000.19710103.x PMC1468110

[95] Wiley DF, Amenta N, Alcantara DA, Ghost D, Kil YJ, Delson E, et al. Evolutionary morphing. In: EEE Visualization, 2005 VIS 05. 2005.

[96] Martin R, Saller K. Lehrbuch der Anthropologie in systematischer Darstellung. 1959. book.

[97] Klingenberg CP. MorphoJ: an integrated software package for geometric morphometrics. Mol Ecol Resour. 2011 3 1;11(2):353–7. 10.1111/j.1755-0998.2010.02924.x 21429143

[98] Adams DC, Collyer ML, Kaliontzopoulou A. Geomorph: Software for geometric morphometric analysis. R package version 3.0. 6. 2018.

[99] Klingenberg CP, McIntyre GS. Geometric morphometrics of developmental instability: analyzing patterns of fluctuating asymmetry with Procrustes methods. Evolution. 1998 10 1;52(5):1363–75. 10.1111/j.1558-5646.1998.tb02018.x 28565401

[100] Mardia KV, Bookstein FL, Moreton IJ. Statistical assessment of bilateral symmetry of shapes. Biometrika. 2000 6 1;87(2):285–300.

[101] Klingenberg CP, Barluenga M, Meyer A. Shape analysis of symmetric structures: quantifying variation among individuals and asymmetry. Evolution. 2002 10 1;56(10):1909–20. 10.1111/j.0014-3820.2002.tb00117.x 12449478

[102] Anderson MJ. A new method for non-parametric multivariate analysis of variance. Aust Ecolology. 2001;26:32–46.

[103] Oksanen J, Blanchet F, Friendly M, Kindt R, Legendre P, McGlinn D et al vegan: Community Ecology Package. R Package Version 25–6 [Internet]. 2019; Available from: https://CRAN.R-project.org/package=vegan

[104] Escoufier Y. Le traitement des variables vectorielles. Biometrics. 1973;29(4):751–60.

[105] Adams DC. Evaluating modularity in morphometric data: challenges with the RV coefficient and a new test measure. Methods Ecol Evol. 2016;7(5):565–72.

[106] Klingenberg CP. Morphometric integration and modularity in configurations of landmarks: tools for evaluating a priori hypotheses. Evol Dev. 2009 Jul;11(4):405–21. 10.1111/j.1525-142X.2009.00347.x 19601974 PMC2776930

[107] Rohlf FJ, Corti M. Use of two-block partial least-squares to study covariation in shape. Syst Biol. 2000 1 12;49(4):740–53. 10.1080/106351500750049806 12116437

[108] Goswami A, Polly PD. The Influence of modularity on cranial morphological disparity in carnivora and primates (Mammalia). PLoS ONE. 2010 3 3;5(3):e9517 10.1371/journal.pone.0009517 20209089 PMC2831076

[109] Klingenberg CP, Marugán-Lobón J. Evolutionary covariation in geometric morphometric data: analyzing integration, modularity, and allometry in a phylogenetic context. Syst Biol. 2013 7;62(4):591–610. 10.1093/sysbio/syt025 23589497

[110] Klingenberg CP. Inferring developmental modularity from morphological integration: analysis of individual variation and asymmetry in bumblebee wings. 2001;157(1):11–23. 10.1086/317002 18707232

[111] Gosse L-A. Essai sur les déformations artificielles du crâne. J.-B. Baillière; 1855. 170 p.

[112] Broca P. Sur les accidents produite par la pratique des déformations artificielles du crâne. Bull Société Anthropol Paris. 1875;10(1):199–204.

[113] Topinard P. Des déformations ethniques du crâne. Rev D’Anthropologie. 1879;2:496–506.

[114] Hrdlička A. Artificial deformations of the human skull, with especial reference to America In: Actas del XVII Congreso Internacional de Americanistas. Buenos Aires; 1910 p. 147–9.

[115] Aichel O. Die künstliche Schädeldeformation. Stuttgart: E. Schweizerbartische Verlagsbuchhandlung; 1932.

[116] Rouse I. The classification of artifacts in archaeology. Am Antiq. 1960;25:313–23.

[117] Manriquez G, Salazar D, Figueroa V, Hartz S, Terberger T. Archaeological fishhooks from the coast of Antofagasta (Atacama Desert, Chile): a geometric morphometric analysis of the Otto Aichel’s fishhooks collection In: Interaktion ohne Grenzen Beispiele archäologischer Forschungen am Beginn des 21 Jahrhunderts. Ed. by Eriksen Berit, Angelika Abegg-Wigg, Ralf Bleile & Ulf Ickerodt. Schleswig, Deutschland: Archäologisches Landesmuseum in der Stiftung Schleswig-Holsteinische Landesmuseen Schloss Gottorf; 2017 p. 958.

[118] Ferros I, Mora M, Obeso I, Jimenez P, Martinez-Insua A. The nasomaxillary complex and the cranial base in artificial cranial deformation: relationships from a geometric morphometric study. Eur J Orthod. 2014;8:1–9. 10.1093/ejo/cju066 25381444

[119] O’Loughlin VD. Effects of Different Kinds of Cranial Deformation on the Incidence of Wormian Bones. Am J Phys Anthropol. 2004;123(2):146–155. 10.1002/ajpa.10304 14730648

[120] Soto-Heim P, Quevedo S. Asymetrie de la base du crane et déformation cranienne. Biométrie Hum Anthropol. 2005;23(3–4):203–11.

[121] Björk A, Björk L. Artificial deformation and cranio-facial asymmetry in ancient Peruvians. J Dent Res. 1964;(3):353–62. 14159038 10.1177/00220345640430030601

[122] Gunz P, Harvati K. The Neanderthal ‘chignon’: variation, integration, and homology. J Hum Evol. 2007 3;52(3):262–74. 10.1016/j.jhevol.2006.08.010 17097133

[123] Scott JH. Muscle growth and function in relation to skeletal morphology. Am J Phys Anthropol. 1957 6 1;15(2):197–234. 10.1002/ajpa.1330150210 13470043

[124] Moss M. The functional matrix In: Kraus B, Riedel R, editors. Vistas in orthodontics. Philadelphia: Lea & Febiger; 1962 p. 85–98.

[125] Frost HM. Bone “mass” and the “mechanostat”: A proposal. Anat Rec. 1987 9 1;219(1):1–9. 10.1002/ar.1092190104 3688455

[126] Pearson OM, Lieberman DE. The aging of Wolff’s “law”: Ontogeny and responses to mechanical loading in cortical bone. Am J Phys Anthropol. 2004 1 1;125(S39):63–99. 10.1002/ajpa.20155 15605390

[127] Ruff C, Holt B, Trinkaus E. Who’s afraid of the big bad Wolff?: “Wolff’s law” and bone functional adaptation. Am J Phys Anthropol. 2006 4 1;129(4):484–98. 10.1002/ajpa.20371 16425178

[128] DiGirolamo DJ, Kiel DP, Esser KA. Bone and skeletal muscle: neighbors with close ties. J Bone Miner Res. 2013 7 1;28(7):1509–18. 10.1002/jbmr.1969 23630111 PMC4892934

[129] Vidarsdottir US, O’Higgins P, Stringer C. A geometric morphometric study of regional differences in the ontogeny of the modern human facial skeleton. J Anat. 2002 9;201(3):211–29. 10.1046/j.1469-7580.2002.00092.x 12363273 PMC1570912

[130] Noback ML, Harvati K, Spoor F. Climate-related variation of the human nasal cavity. Am J Phys Anthropol. 2011 8 1;145(4):599–614. 10.1002/ajpa.21523 21660932

[131] Nicholson E, Harvati K. Quantitative analysis of human mandibular shape using three-dimensional geometric morphometrics. Am J Phys Anthropol. 2006 11 1;131(3):368–83. 10.1002/ajpa.20425 16617436

[132] Smith HF. The role of genetic drift in shaping modern human cranial evolution: a test using microevolutionary modeling. Int J Evol Biol. 2011 3 3;2011:e145262. 10.4061/2011/145262 PMC3065169 21461369

