# Supplementary material for: Morphological consequences of artificial cranial deformation: Modularity and integration
Source: PLoS One. 2020 Jan 24;15(1):e0227362. doi: 10.1371/journal.pone.0227362 (PMC6980596; doi:10.1371/journal.pone.0227362)
Supplement: S1 Fig — CR coefficients obtained from permutation tests (999 rounds) of alternative partitions of a) the complete dataset, b) antero-posterior deformed skulls, c) non-deformed crania and d) the oblique sample, with the observed CR coefficients designated by a red arrow. Both the symmetric and asymmetric components of shape variation were analyzed. (PDF) [file pone.0227362.s004.pdf]

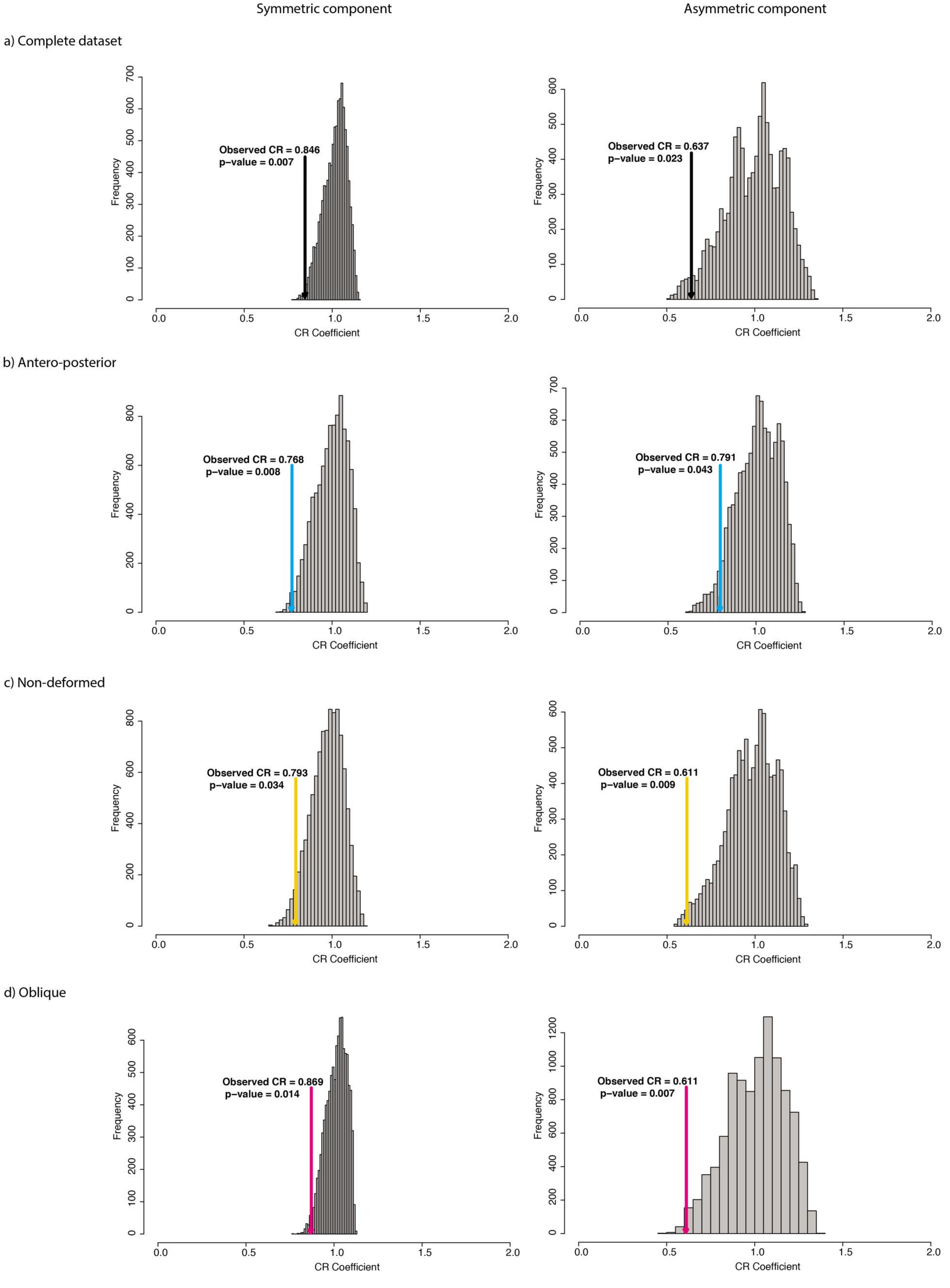

Symmetric component
Asymmetric component
a) Complete dataset
Observed CR = 0.846
p–value = 0.007
Observed CR = 0.637
p–value = 0.023
b) Antero-posterior
Observed CR = 0.768
p–value = 0.008
Observed CR = 0.791
p–value = 0.043
c) Non-deformed
Observed CR = 0.793
p–value = 0.034
Observed CR = 0.611
p–value = 0.009
d) Oblique
Observed CR = 0.869
p–value = 0.014
Observed CR = 0.611
p–value = 0.007
Frequency
CR Coefficient
