# Supplementary figures and images for: Morphological consequences of artificial cranial deformation: Modularity and integration
Source: PLoS One. 2020 Jan 24;15(1):e0227362. doi: 10.1371/journal.pone.0227362 (PMC6980596; doi:10.1371/journal.pone.0227362)

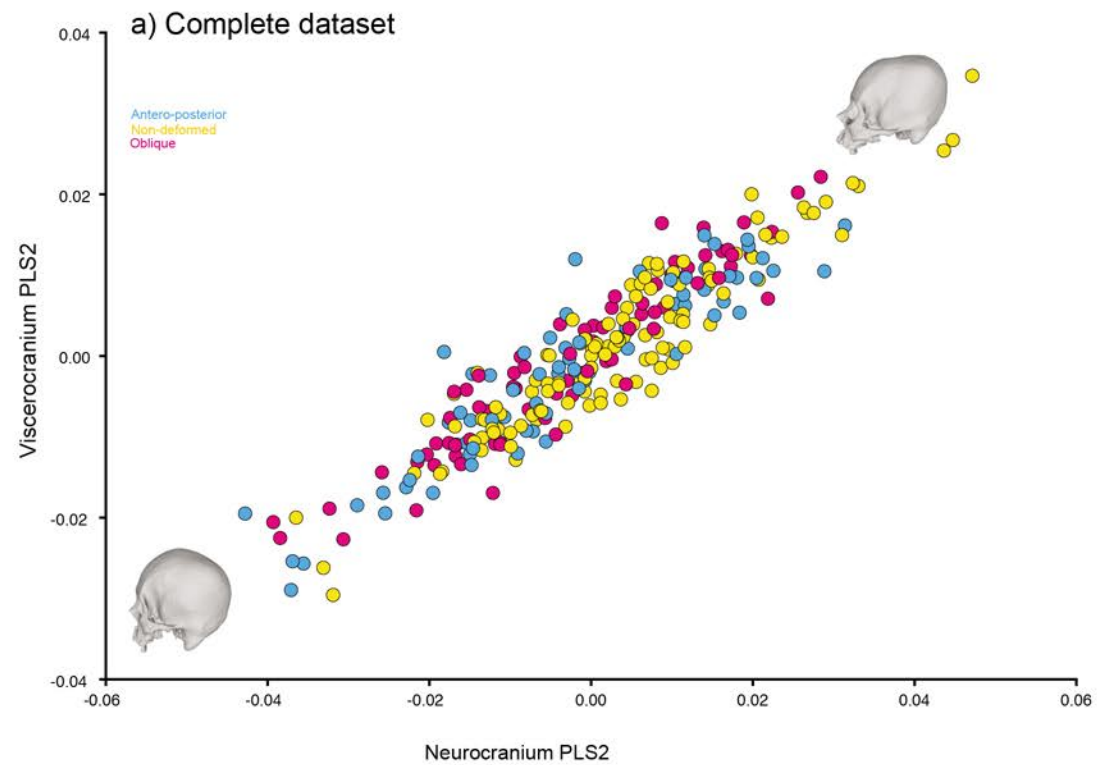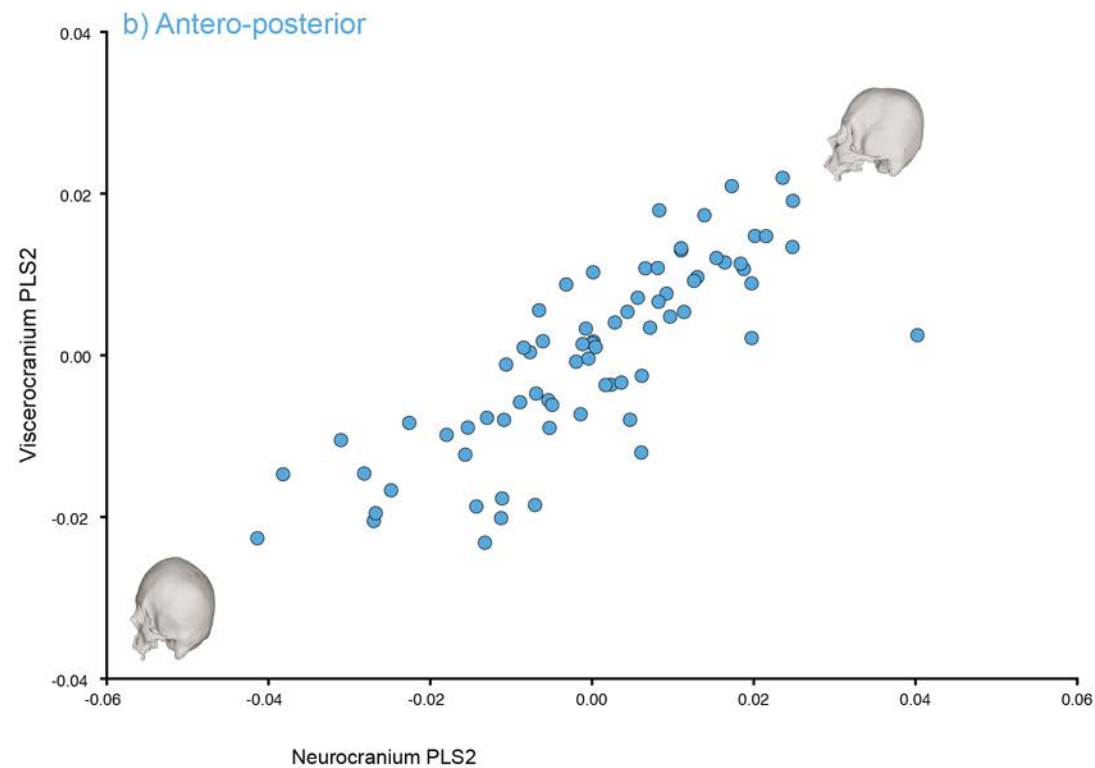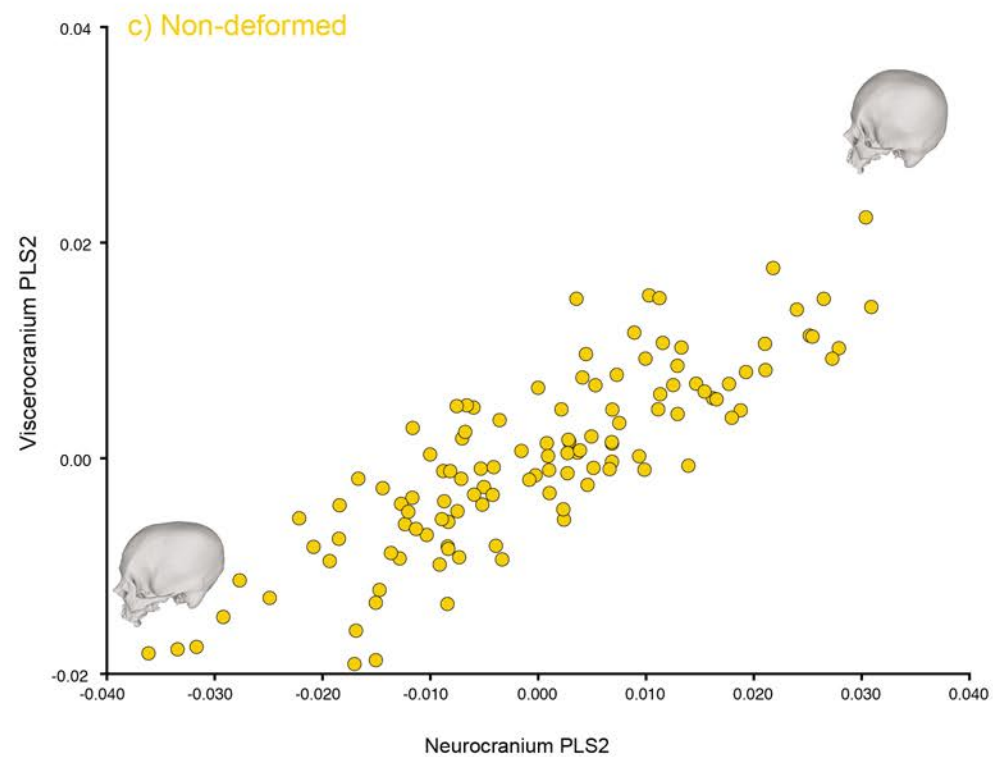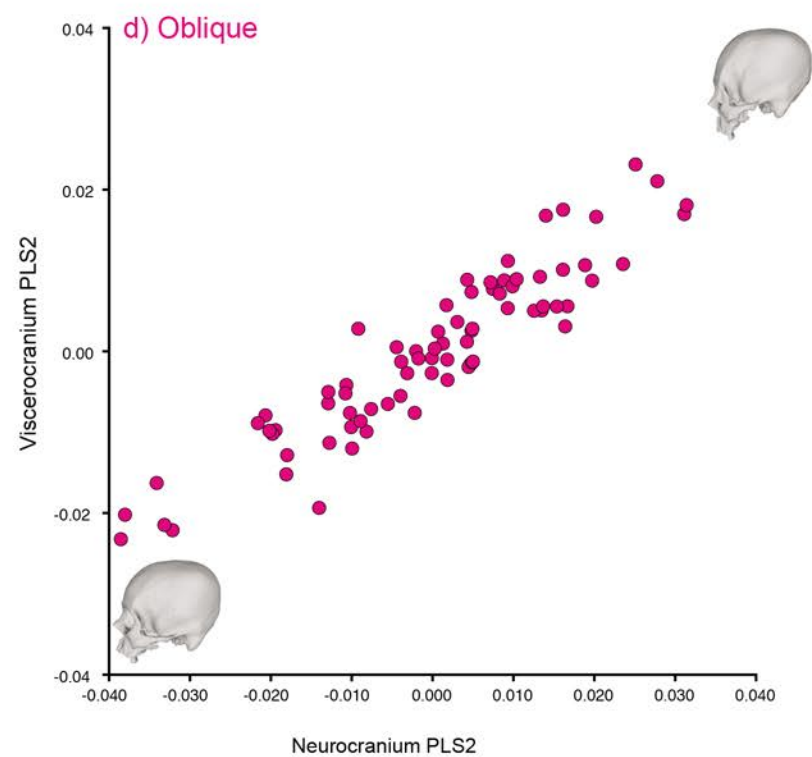

Supplement: S2 Fig — a) the Complete dataset, as welll as for the different cranial categories under analysis b) Antero-posterior; c) Non-deformed and; d) Oblique. (PDF) [file pone.0227362.s005.pdf]
